# ﻿A taxonomic revision of the genus *Alexeter* Förster (Hymenoptera, Ichneumonidae, Ctenopelmatinae, Mesoleiini) from Taiwan, with descriptions of six new species

**DOI:** 10.3897/zookeys.1250.156835

**Published:** 2025-09-02

**Authors:** Hsuan-Pu Chen, Chia-Lung Huang, Shiuh-Feng Shiao

**Affiliations:** 1 Department of Entomology, National Taiwan University, No.1, Sec. 4, Roosevelt Road, Taipei 10617, Taiwan National Taiwan University Taipei Taiwan; 2 Fujian Key Laboratory on Conservation and Sustainable Utilization of Marine Biodiversity, Fuzhou Institute of Oceanography, College of Geography and Oceanography, Minjiang University, Fuzhou 350108, China Minjiang University Fuzhou China

**Keywords:** Darwin wasps, high elevation, molecular phylogeny, species delimitation, taxonomy

## Abstract

The genus *Alexeter* Förster, 1869 is first recorded from Taiwan. One previously described species, *A. shakojiensis* Uchida, 1930, is newly recorded from Taiwan. Six new species, *A. flavomaculatus***sp. nov.**, *A. hsiaoae***sp. nov.**, *A. mediolobus***sp. nov.**, *A. monticola***sp. nov.**, *A. pseudozangicus***sp. nov.**, and *A. rufispeculus***sp. nov.**, are described and can be distinguished from their congeners primarily based on color pattern, mandibular teeth, propodeal carinae, fore wing length and venation, ocellar and first metasomal tergite measurements, and flagellomere counts. Illustrations of the male genitalia and a diagnostic key to the Taiwanese *Alexeter* species are provided. DNA-based species delimitations are provided as supporting evidence for four new species. In a COI-based phylogeny sampling 31 operative taxonomic units of *Alexeter* and similar genera, the genus *Alexeter* was not resolved as a monophyletic group. Additionally, the *COI* barcode showed limitations in distinguishing some *Alexeter* morphospecies, indicating the need for further evaluation of *COI*-based species delimitation in this genus.

## ﻿Introduction

The tribe Mesoleiini Thomson, 1883 represents a diverse group of Darwin wasps (Ichneumonidae) within the subfamily Ctenopelmatinae, comprising approximately 600 described species (Yu et al. 2016). Members of this tribe are koinobiont endoparasitoids of larval Symphyta and are predominantly distributed in the Holarctic regions, with fewer species found in the Neotropical and Oriental regions (Townes 1970; Gauld 1997; Luo et al. 2019; Sheng et al. 2020). Currently, 27 genera are recognized within Mesoleiini (Yu et al. 2016; Kasparyan 2020), but clear boundaries and monophyly of these genera are still pending since delimitation of some genera is vague (Townes 1970; Gauld 1997).

*Alexeter* Förster, 1869 comprises 38 valid species, with 17 species found in Asia (Yu et al. 2016; Sun et al. 2019; Sheng et al. 2020; Li and Sun 2022). It is known for parasitizing Tenthredinoidea, although little is known about the biology and host repertoire of most species (e.g., Györfi 1947; Hinz 1961; Gauld 1997; Aubert 2000). Similar to other mesoleiine genera, *Alexeter* exhibits its highest species diversity in the Holarctic regions, with only six species recorded in mountainous areas of the Neotropical and Oriental regions (Gauld 1997; Yu et al. 2016; Sheng et al. 2020; Li and Sun 2022). Since Ctenopelmatinae and their main hosts, symphytans, exhibit similar distribution patterns of biodiversity, the biogeography of these wasps may be associated with their hosts and could have involved tropical lineages spreading from the northern temperate regions along mountain ranges (Malaise 1945; Smith 2011; Reshchikov 2015; Reshchikov et al. 2022).

Taiwan is a continental island located in subtropical East Asia and characterized by its mountainous terrain. Since high elevation mountains in tropical or subtropical regions often exhibit climatic conditions similar to those in temperate regions, Taiwan plays a transitional role in hosting fauna that comprises both Eastern Palearctic and Oriental taxa (Sarmiento 1986; Päckert et al. 2012; He et al. 2018; Gąsiorek et al. 2021). Currently, only one mesoleiine species, *Barytarbes lalashanensis* (Kusigemati, 1990), has been recorded in Taiwan (Kusigemati 1990). Given the Holarctic distribution of most mesoleiine Darwin wasps, the unique biogeographical features of Taiwan, and the limited investigation of Ichneumonidae in its high elevation areas, it is plausible that the Taiwanese fauna of Mesoleiini has been significantly underestimated. Therefore, the presence of the genus *Alexeter* is expected in the mountainous areas of Taiwan.

This study aims to record the genus *Alexeter* from Taiwan for the first time and revise the Taiwanese species based on multiline evidence, combining morphological comparisons and DNA-based species delimitation using the partial sequences of cytochrome c oxidase subunit I (*COI*) gene (COI-5P region). The monophyly of the genus *Alexeter* is also tested based on the *COI*-based molecular phylogeny.

## ﻿Materials and methods

### ﻿Institutional abbreviations

The specimens/photos examined were deposited in the following institutions:

**ANSDU** Academy of Natural Science of Drexel University, Philadelphia, PA, USA

**AEIC** American Entomological Institute, Utah State University, Logan, UT, USA

**NHRS** Naturhistoriska Riksmuseet, Stockholm, Sweden

**NMNS** National Museum of Natural Science, Taichung, Taiwan

**TARI** Taiwan Agriculture Research Institute, Taichung, Taiwan

**TFRI** Taiwan Forestry Research Institute, Taipei, Taiwan

**SEHU** Laboratory of Systematic Entomology, Hokkaido University, Sapporo, Hokkaido, Japan

**KPMNH** Kanagawa Prefectural Museum of Natural History, Odawara, Kanagawa, Japan

**GSFGPM** General Station of Forest and Grassland Pest Management, National Forestry and Grassland Administration, Shenyang, China

### ﻿Taxon sampling

A total of 122 Taiwanese specimens of *Alexeter* were collected from middle to high elevation mountains (elevation 1200–3450 m) of Taiwan by Malaise trap, sweeping, or light trap. For comparison with the newly described species, type specimens, voucher specimens, and photos of 15 of the 38 known *Alexeter* species, as well as two species previously classified within *Alexeter*, were examined.

The examined comparative material is as follows: *A. nebulator* (Thunberg, 1822) (2♂♂, 2♀♀, KPMNH); *A. daisetsuzanus* Uchida, 1930 (holotype, ♀, SEHU; 1♂, 1♀, KPMNH); *A. dorogawaensis* Uchida, 1934 (holotype, ♂, SEHU); *A. gracilentus* (Holmgren, 1857) (lectotype, ♀, NHRS-HEVA000004432, examined by photos); *A. fallax* (Holmgren, 1857) (syntype, ♀, NHRS-HEVA000004428, examined by photos); *A. improbus* (Holmgren, 1857) (lectotype, ♀, NHRS-HEVA000004868, examined by photos); *A. napaeus* (Holmgren, 1857) (currently synonymized with *A. multicolor* (Gravenhorst, 1829)) (lectotype, ♀, NHRS-HEVA000004955, examined by photos); *A. obscuricolor* Heinrich, 1953 (holotype, ♀, AEI, examined by photos); *A. difficilis* (Davis, 1897) (lectotype, ♂, ANSDU 4391, examined by photos); *A. innoxius* (Cresson, 1879) (lectotype, ♂, ANSDU 1305, examined by photos); *A. luteifrons* (Cresson, 1868) (lectotype, ♂, ANSDU 1400, examined by photos); *A. notatus* Davis, 1897 (lectotype, ♂, ANSDU 4409, examined by photos); *A. scapularis* (Cresson,1868) (lectotype, ♂, ANSDU 1382; examined by photos); *A. clavator* (Müller, 1776) (1♀, GSHGPM, examined by photos; 1♀, examined by photos available at https://www.bioimages.org.uk/html/r167651.htm); *A. segmentarius* (Fabricius, 1787) (1♀, examined by photos available at https://www.bioimages.org.uk/html/r169606.htm); *Perispuda angularis* (Uchida, 1952) (holotype, ♀, SEHU, examined by photos); *B. compos* (Davis, 1897) (lectotype, ♂, ANSDU 4408, examined by photos); and *B. lalashanensis* (Kusigemati, 1990) (3♀♀, TARI). The other species of this genus were compared based on the original descriptions and photographs in the literatures (e.g., Gauld 1997; Sun et al. 2019; Sheng et al. 2020; Li and Sun 2022).

### ﻿Morphological examination and description

**Figure 1.**
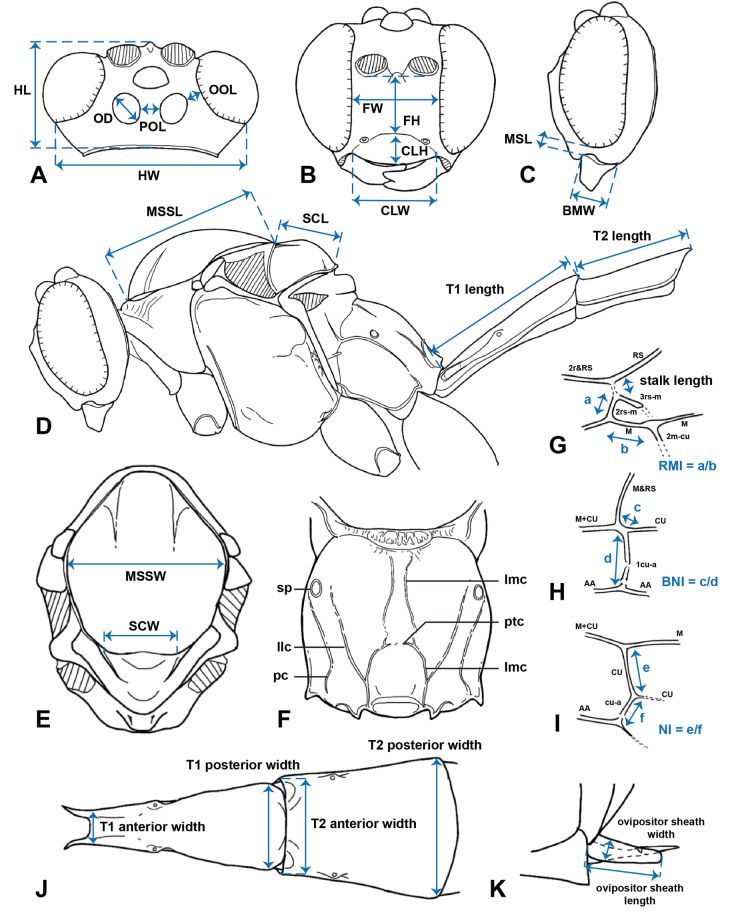
Schematic drawing of the methods of measurements and partial morphological terminology of *Alexeter* used in this study. **A.** Head in dorsal view; **B.** Head in anterior view; **C.** Head in lateral view; **D.** Mesosoma and metasomal tergites 1 and 2 in lateral view; **E.** Mesoscutum and scutellum in dorsal view; **F.** Propodeum in dorsal view; **G.** Fore wing areolet; **H.** Fore wing vein M&RS and 1cu-a; **I.** Hind wing nervellus; **J.** Metasomal tergites 1 and 2 in dorsal view; **K.** Ovipositor sheath and ovipositor. Abbreviations: BMW, basal mandibular width; BNI, basal-nervulus index of fore wing; CLH, height of clypeus; CLW, width of clypeus; FH, height of face; FW, maximum width of face; HL, length of head in dorsal view; HW, width of head in dorsal view; MSL, length of malar space; MSSL, length of mesoscutum; MSSW, maximum width of mesoscutum; NI, nervellar index of hind wing; OD, ocellar diameter; OOL, ocello-ocular line; POL, postero-ocellar line; RMI, radius-media index of fore wing; SCL, length of scutellum; SCW, anterior width of scutellum; T, metasomal tergite; lmc, lateromedian longitudinal carina; llc, lateral longitudinal carina; pc, pleural carina; ptc, posterior transverse carina; sp, spiracles.

Morphological terminology is as per Broad et al. (2018). The methods of measurement and abbreviations mostly follow Kikuchi and Konishi (2021) and illustrate as Fig. 1. The abbreviations and their definitions used in this study are listed as follows: **OOL** = ocello-ocular line, minimum distance between lateral ocellus and eye; **POL** = postero-ocellar line, minimum distance between lateral ocelli; **OD** = ocellar diameter, diameter of lateral ocellus; **HL** = length of head in dorsal view; **HW** = width of head in dorsal view, measured at the intersection between temple and posterior margin of eyes; **BMW** = basal mandibular width in lateral view; **MSL** = length of malar space, minimum distance from eye to mandibular base in lateral view; **FH** = height of face, measured by distance between the lines connecting lower margins of each antennal sockets and upper margin of each anterior tentorial pits in anterior view; **FW** = maximum width of face, measured by minimum distance between eyes in anterior view; **CLH** = height of clypeus, measured from groove separating face and clypeus to ventral margin of clypeus; **CLW** = width of clypeus; **MSSL** = length of mesoscutum, measured in lateral profile; **MSSW** = maximum width of mesoscutum, measured in dorsal view; **SCL** = length of scutellum, measured in lateral profile; **SCW** = anterior width of scutellum; **RMI** = radius-media index of fore wing, length of 2rs-m/length of M between 2rs-m and 2m-cu; **BNI** = basal-nervulus index of fore wing, distance between M&RS and 1cu-a/length of 1cu-a; **NI** = nervellar index of hind wing, length of hind wing CU between M and cu-a/length of cu-a; **T** = metasomal tergite, length measured in lateral view; anterior width measured as the minimum width anteriorly in dorsal view; posterior width measured as the maximum width posteriorly in dorsal view; **S** = abdominal sternites.

The measurements and counts in parentheses represent the holotype measurement, mean ± standard deviation. However, the mean and standard deviation are not calculated for the counts. The size of ocelli is defined as normal (OOL/OD ratio > 1.0) and large (OOL/OD ratio < 1.0). Description of the cuticular microsculpture as per Eady (1968). Terminology of male genitalia as per Dal Pos et al. (2023). Specimens were examined and measured by microscope LEICA S8APO (Leica Microsystems, Germany) with XFCAM autofocus CCD (Jet measurements, Taiwan). Photographs were taken by LEICA DMC5400 adjacent to LEICA Z16 APO with auto-stacking system LAS V4.13 (Leica Microsystems, Wetzler, Germany). All line drawings were created using Procreate (Savage Interactive, Australia) via iPad Air 4 (Apple Inc., USA). The Latin term ibidem, meaning “same as previous except as follows” and abbreviated as ‘ibid’, was used for compressing the locality information of materials examined.

### ﻿Molecular data

Total genomic DNA was extracted from a right mid leg of the specimen by DNeasy Blood and Tissue Kit (Qiagen, Düsseldorf, Germany) by following the manufacturer’s protocol but eluting in 100 μl volume. Partial sequences of *COI* were amplified using the primer pairs LCO1490/HCO2198 (Folmer 1994) or Fol_Ich_F/Fol_Ich_R (Chen et al. 2023). If co-amplification of endosymbionts occurred, C1-J-1718/C1-N-2329 (Rousse et al. 2016) were then used for amplifying the sequences downstream of the *COI*-5P region for ~200 bp.

PCR conditions were 4 min at 95 °C as an initial denaturation, and 35 cycles of 30 s at 95 °C of denaturation, 1 min at 45 °C of annealing, and 45 s at 72 °C of extension, then a final extension at 72 °C for 10 mins for LCO1490/HCO2198 and Fol_Ich_F/Fol_Ich_R. For C1-J-1718/C1-N-2329, the condition was modified with 35 cycles of 30 s at 94 °C of denaturation, 30 s at 50 °C of annealing, and 45 s at 72 °C of extension, and 7 mins at 72 °C of final extension. Each PCR was conducted in a 15 µL volume containing 5.1 µL sterile distilled water, 0.6 µL of each primer (10 µM), 7.5 µL of GoTaq® Green Master Mix (Promega, Madison, WI, USA) and 1.2 µL of DNA template. All PCR products were purified and sequenced at Biotech company Tri-I Biotech Inc. (Taipei, Taiwan). Sequences were edited by Codoncode Aligner v. 10.0.2 (CodonCode Corporation, Dedham, MA, USA), preliminarily identified by BLAST (Altschul et al. 1990), then aligned using MAFFT V7 (Katoh et al. 2019) and translated into amino acids for checking stop codons in MEGA11 (Tamura et al. 2021). All sequences were uploaded to GenBank (NCBI, Nation Center for Biotechnology Information).

### ﻿Molecular phylogeny and species delimitation

For phylogenetic analysis, 46 *COI* sequences of *Alexeter* from BOLD systems, consisting of 20 operational taxonomic units (OTUs) delimited by clusters of Barcode Index Numbers (BINs), were included as the ingroup. Additionally, ten *COI* sequences forming seven OTUs were chosen from morphologically similar genera: *Barytarbes* Förster, 1869 (*B. flavicornis* (Thomson, 1892), *B. honestus* (Cresson, 1868)), *Campodorus* Förster, 1869 (*Cam. melanogaster* (Holmgren, 1857), *Cam. dorsalis* (Gravenhorst, 1829)), and *Lagarotis* Förster, 1869 (*L. debitor* (Thunberg, 1824), *L. simulator* Heinrich, 1952). One sequence of *Ctenopelma nigrum* Holmgren, 1857 was chosen as the outgroup (Townes 1970; Wharton 2015). The phylogenetic tree was rooted by *Cte. nigrum*.

The maximum likelihood phylogenetic tree was reconstructed by the program IQ-TREE 1.6.12 (Nguyen et al. 2015) through the web server W-IQ-TREE (Trifinopoulos et al. 2016) (available at http://iqtree.cibiv.univie.ac.at/). Nodal support was assessed with ultrafast bootstrap approximation (UFBoot) (Minh et al. 2013) and SH-like approximate likelihood ratio test (SH-aLRT) (Guindon et al. 2010) based on default parameter settings in the program. The nodes with SH-aLRT ≥ 80% and UFBoot ≥ 95% were considered strongly supported nodes.

Assemble Species by Automatic Partitioning (ASAP) (Puillandre et al. 2020) and Bayesian-based Poisson Tree Processes (bPTP) (Zhang et al. 2013) were used for DNA-based species delimitation. The ASAP was conducted with *COI* dataset under the K2P distance, and bPTP was conducted based on an unrooted *COI* gene tree. The analyses were performed using the web interfaces (ASAP: https://bioinfo.mnhn.fr/abi/public/asap/ and bPTP: https://species.h-its.org/) with default parameter settings.

## ﻿Results

### ﻿Molecular phylogeny

The *COI* dataset used for phylogenetic reconstruction and species delimitation analyses comprised a total of 67 sequences, including 46 sequences of *Alexeter* obtained from BOLD, 11 outgroup sequences, and ten sequences of four species of Taiwanese *Alexeter*. The dataset has an alignment length of 809 base pairs (bp), average length 645.6 bp, GC content of 26.5%, 279 bp of variable sites, and 231 bp of parsimony-informative sites. Sequence information and specimen data are provided in Suppl. material 1, and the dataset (Fasta file and the partition scheme in the Nexus file) is provided in Suppl. material 2.

**Figure 2.**
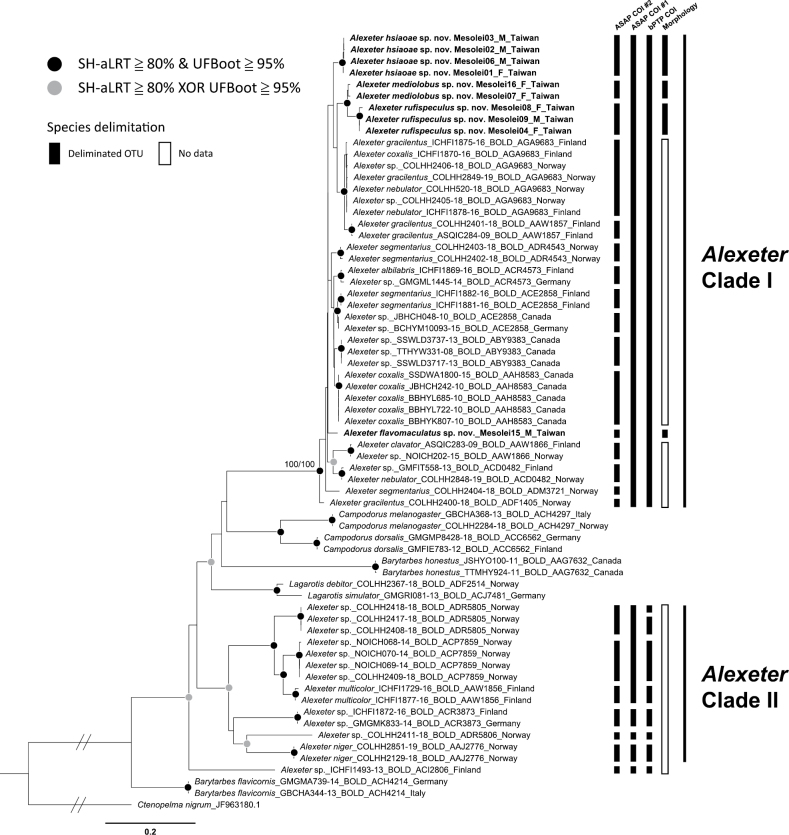
Maximum-likelihood phylogenetic tree of the species of *Alexeter* Förster, 1869 and its morphologically similar genera based on the *COI* dataset (809 bp; HKY+F+I+G4 in 1–809\3 and 2–809\3, TIM3+F+G4 in 3-809\3), with species delimitation results and clades of *Alexeter* labeled on the right side of the tree. Taxon name in bold indicated the new species described herein. Branch lengths of the tree are proportional to the inferred number of nucleotide substitutions per site except for the root *Ctenopelma nigrum*. Circles on the nodes indicate different SH-aLRT/UFBoot values. Nodal support with an SH-aLRT value of < 80% and a UFBoot value of < 95% is not shown. Abbreviations: SH-aLRT, SH-like approximate likelihood ratio test; UFBoot, ultrafast bootstrap approximation; XOR, one or the other but not both; M, male; F, female.

The *COI*-based maximum likelihood phylogenetic tree, presented in Fig. 2, shows that the OTUs identified as the genus *Alexeter* do not form a monophyletic group. The *Alexeter*OTUs form three lineages, *Alexeter* Clade I, *Alexeter* Clade II, and “*Alexeter* sp.” (BOLD:ACI2806) (Fig. 2). Clade I is strongly supported (UFBOOT/SH-aLRT = 100/100) and exhibits relatively short branch lengths (Fig. 2). All sampled Taiwanese species are included within Clade I. The outgroups, except for the root *Ctenopelma nigrum*, are interspersed between the three *Alexeter* lineages. *Barytarbes flavicornis* represents the first-splitting lineage among the sampled OTUs, while the two *Campodorus*OTUs form the sister group of *Alexeter* Clade I. Additionally, *Barytarbes honestus* and the lineage containing two *Lagarotis*OTUs diverge from the clade comprising *Campodorus* and *Alexeter* Clade I (Fig. 2).

### ﻿Species delimitation

Focusing on the sampled *Alexeter*OTUs, the suboptimal partition of ASAP (ASAP #2 in Fig. 2) interpreted all morphospecies and BINs as independent species (Fig. 2), with a COI threshold distance of 0.008414 between putative species. On the other hand, the optimal partition of ASAP (ASAP #1) and bPTP merged the morphospecies and BINs in Clade I into a single species (Fig. 2). Additionally, in Clade II, the best partition of ASAP merged the BINsBOLD:ADR5805, BOLD:ACP7859, and BOLD:AAW1856 into one species, while the bPTP results split BIN BOLD:ADR5805 into two putative species (Fig. 2). Four Taiwanese species with molecular data form monophyletic groups respectively and can be separated by their morphological characters, as presented in the Taxonomy section below.

### ﻿Taxonomy

Based on the thorough examination of specimens collected during high mountain expeditions and from museum collections, comparisons with type specimens, and *COI*-based species delimitation, one known species, *A. shakojiensis* Uchida, 1930 and six new species of *Alexeter* are recognized and described from Taiwan. Partial sequences of the *COI* gene for four of the seven species and an identification key to Taiwanese species are provided herein. Additionally, tables including detailed measurements of Taiwanese species and comparison between yellowish- or reddish-brown species of *Alexeter* of the world are provided in Suppl. material 3.

**Order Hymenoptera Linnaeus, 1758**

**Family Ichneumonidae Latreille, 1802**

**Subfamily Ctenopelmatinae Förster, 1869**

**Tribe Mesoleiini Thomson, 1883**

#### ﻿Genus Alexeter

Taxon classificationAnimaliaHymenopteraIchneumonidae

Förster, 1869

DC23034A-8D19-52D2-BEED-552AEFA0D9C5

Alexeter Förster, 1869: 212. Type species: Mesoleptus ruficornis Gravenhorst, 1829. Designated by Viereck 1914: 7.
Adranes Förster, 1869: 205. Type species: Tryphon multicolor Gravenhorst, 1829. Designation by Townes et al. 1965: 255. Synonymized by Townes et al. 1965: 255. Preoccupied by Adranes Leconte, 1850.
Zemiophron Förster, 1869: 206. Type species: Mesoleius laevissimus Strobl, 1903. Designation by Perkins 1962: 464. Synonymized by Perkins 1962: 464.

##### Diagnosis.

According to Townes (1970), Gauld (1997), and Wharton (2015), this genus can be distinguished from other genera of Mesoleiini by the combination of the following characters: sculpture of head and mesosoma generally matt, granulate or minutely coriaceous; mandibular teeth usually equal in length (lower tooth longer in few Holarctic species and all known Neotropical species); notaulus usually distinct at least near anterior margin of mesoscutum; fore wing areolet usually present; fore wing 1cu-a usually opposite to M&RS; T1 long and slender, usually longer than 2.5 × its posterior width; T1 with latero-median carinae absent, dorsal lateral carina distinct, and glymma always distinct; lateromedian longitudinal carinae of propodeum distinct posteriorly and forming an area petiolaris with median part of posterior transverse carinae.

This genus is similar to *Barytarbes*, as some *Alexeter* species share the absence of anterior and median portions of the lateromedian longitudinal carinae on the propodeum and have mandibles with the lower tooth longer than the upper. However, *Barytarbes* typically features a shorter and broader T1 and a propodeum with weaker lateromedian longitudinal carinae in the posterior part. Additionally, some *Barytarbes* species lack a glymma on the T1, distinguishing them from *Alexeter*.

Townes (1970) also mentioned that some *Alexeter* species are challenging to separate from genera such as *Campodorus*, *Lagarotis*, and *Alcochera*. Generally, *Alexeter* can be separated from *Campodorus* by having a clypeus with sharp, truncate or rounded ventral margin, flat in lateral view or with subventral transverse ridge or median elevation (clypeus strongly bulging medially and subapically with bilobed ventral margin in *Campodorus*), and areolet usually present (always absent in *Campodorus*). It can also be separated from *Lagarotis* in having mesopleuron granulate or minutely coriaceous (heavily sculptured, e.g., minutely rugose, in *Lagarotis*). Lastly, it can be separated from *Alcochera* by the mandible with teeth usually equal in length (lower tooth always longer than upper tooth in *Alcochera*), and notaulus distinct at least near anterior margin (weak in *Alcochera*).

##### Remarks.

In this study, we employed two criteria to determine the generic placement of the Taiwanese mesoleiines: morphological characters closely aligned with the generic definition proposed by Townes (1970) and the notes on Wharton’s website (summarized in the Diagnosis above); and when applicable, specimens with *COI* sequences similar to those identified as *Alexeter* in online databases such as BOLD Systems and GenBank.

The characters distinguishing this genus from morphologically similar genera – such as the length of the mandibular teeth, the length and strength of the notauli on the mesoscutum, the presence of a fore wing areolet, the propodeal carinae, and the length of metasomal T1 – exhibit considerable variation. Therefore, the generic boundary of *Alexeter* and other mesoleiine genera is ambiguous, challenging the generic position of some species (Townes 1970; Wharton 2015). The vague generic boundaries may indicate that the currently defined *Alexeter* is non-monophyletic, which is preliminarily supported by the *COI*-based phylogeny reconstructed in this study (Fig. 2). It suggests the necessity of a more comprehensive taxonomic revision of *Alexeter* and similar genera.

### ﻿Key to Taiwanese species of *Alexeter* Förster, 1869

**Table:** 

1	Head (at least vertex and temple) and mesosoma generally black (Figs 3A, 4A, 12C); metasoma entirely reddish-brown or black with yellow marking on tergites (Figs 3A, 4A)	**2**
–	Head, mesosoma, and metasoma generally reddish-brown or yellowish-brown, if blackish-brown, not as above (Figs 5A, 6A, 7A, 8A, 9A, 12F, I–K)	**3**
2	Fore wing length usually longer than 10.0 mm (9.7–11.9 (10.7 ± 0.77) mm); MSSL/MSSW = 1.3–1.5; propodeum with lateromedian longitudinal carinae complete (Fig. 3E); mesosoma without yellow markings (Fig. 3A); scutellum and whole metasoma reddish-brown (Fig. 3A, D)	***Alexeter shakojiensis* Uchida, 1930**
–	Fore wing length not longer than 9.0 mm (7.2–8.3 (7.7 ± 0.45) mm); MSSL/MSSW = 1.2–1.3; propodeum with lateromedian longitudinal carinae absent or vestigial on anterior and median portions (Fig. 4E); mesosoma with yellow markings on latero-anterior corners of mesoscutum and ventral area of mesopleuron (Figs 4A, D, 12B, C); scutellum yellow (Figs 4D, 12B); metasoma black with yellow markings on tergites (Fig. 4F)	***Alexeter flavomaculatus* Chen, Huang & Shiao, sp. nov.**
3	Head, mesosoma, and metasoma generally blackish-brown to reddish-brown, not yellowish-brown (Figs 8A, 9A, 12F, I–K); mesoscutum with yellow or yellowish-white markings on latero-anterior corners at least in male (Figs 8D, 12E, H, J)	**4**
–	Head, mesosoma, and metasoma generally brown to yellowish-brown (Figs 5A, 6A, 7A); mesoscutum with brown or blackish-brown longitudinal stripe (Figs 5D, 6D, 7D)	**5**
4	Clypeus rounded on ventral margin (Fig. 8C, 12D); mandible with lower tooth slightly longer than upper tooth (at least females) (Fig. 8C); fore wing without areolet (Fig. 10F); propodeum with lateromedian longitudinal carinae complete (Fig. 8E); T1 2.3–3.0 × as long as its posterior width; male gonostyle broad (Fig. 11Q); general body color reddish-brown (female) or blackish-brown (male) (Figs 8, 12D–F); mesoscutum with yellow markings on latero-anterior corners in both sexes (Figs 8D, 12E)	***Alexeter pseudozangicus* Chen, Huang & Shiao, sp. nov.**
–	Clypeus truncate on ventral margin (Fig. 9C, 12G); mandible with teeth equal in length (Fig. 9C, 12G); fore wing with areolet (Fig. 10G); propodeum with lateromedian longitudinal carinae absent on anterior and median portions (Fig. 9E); T1 3.0–4.3 × as long as its posterior width; male gonostyle not broad (Fig. 11O); general body color ranges from reddish-brown to blackish-brown (Figs 9, 12H–K); mesoscutum with yellow or yellowish-white markings on latero-anterior corners in males only (Fig. 12H, J)	***Alexeter rufispeculus* Chen, Huang & Shiao, sp. nov.**
5	Flagellum with 40–43 segments in female; T1 2.5–3.1 × as long as its posterior width; propodeum with area petiolaris almost closed anteriorly (Fig. 6E); mesoscutum with one longitudinal brownish stripe on median lobe (Fig. 6D)	***Alexeter mediolobus* Chen, Huang & Shiao, sp. nov.**
–	Flagellum with 46–52 segments in female; T1 3.1–3.9 × as long as its posterior width; propodeum with area petiolaris opened anteriorly (Figs 5E, 7E); mesoscutum with three longitudinal brownish stripes on lobes (Figs 5D, 7D)	6
6	Gena, fore and mid coxae yellowish-white (Fig. 5A); vertex brown (Fig. 5B); mesoscutum yellowish-white, with three longitudinal blackish-brown stripes on lobes distinct (Fig. 5D); flagellum with 46 segments in female (all specimens); OOL/OD = 0.7–0.9; distal hamuli 5–6; habitats known from areas above 3000 m (3000–3450 m)	***Alexeter hsiaoae* Chen, Huang & Shiao, sp. nov.**
–	Gena, fore and mid coxae yellowish-brown or orange (Fig. 7A); vertex yellowish-brown or orange (Fig. 7B); mesoscutum yellowish-brown or orange, with three longitudinal brownish stripes on lobes (not as distinct as *A. hsiaoae* sp. nov.) (Fig. 7D); flagellum usually exceed 46 segments in female (46–52, but only one specimen with 46 segments); OOL/OD = 0.5–0.7; distal hamuli 6–9; habitats known from areas below 3000 m (2300–2800 m)	***Alexeter monticola* Chen, Huang & Shiao, sp. nov.**

#### ﻿ Alexeter shakojiensis

Taxon classificationAnimaliaHymenopteraIchneumonidae

Uchida, 1930

7D012608-2394-5AB6-88AB-EC2F69CB8984

**Figure 3.**
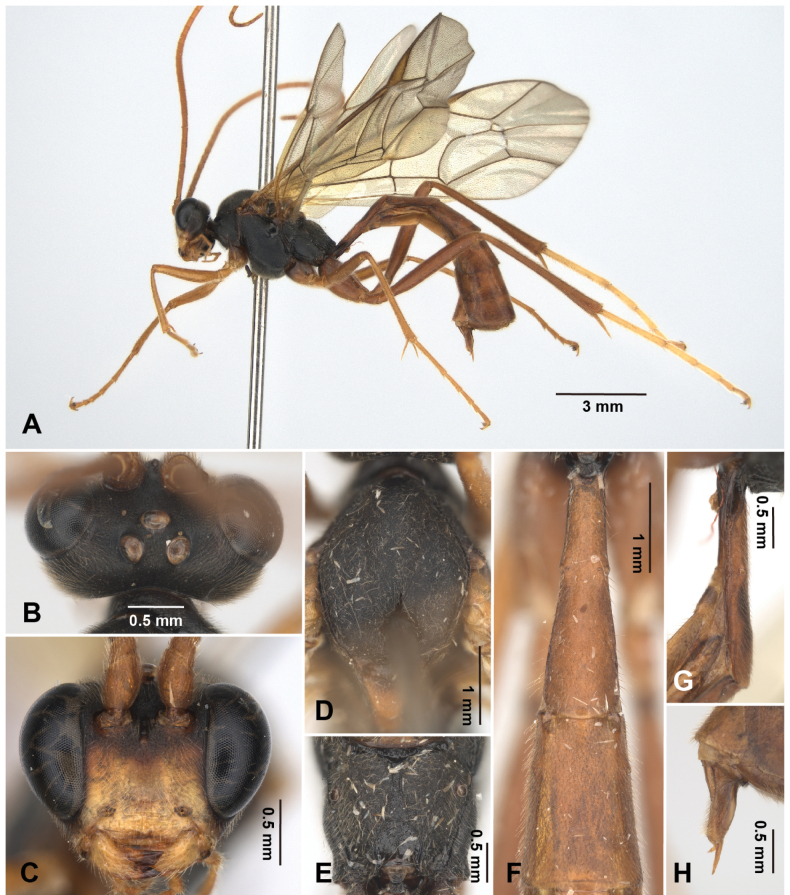
*Alexeter shakojiensis* Uchida, 1930, female (TARI). **A.** Lateral habitus; **B.** Head in dorsal view; **C.** Head in anterior view; **D.** Mesoscutum in dorsal view; **E.** Propodeum in dorsal view; **F.** Metasomal tergites 1 and 2 in dorsal view; **G.** Metasomal tergite 1 in lateral view; **H.** Ovipositor.

Figs 3A–H, 10A, 11A–C; Suppl. material 3 Chinese vernacular name: 朝鮮亞力姬蜂 / 朝亞力姬蜂

Alexeter shakojiensis Uchida, 1930: 292.
Alexeter shakojiensis – Kim 1955: 491; Townes et al. 1965: 257; Sun et al. 2019: 86; Sheng et al. 2020: 156; Li and Sun 2022: 99.
Mesoleius shakojiensis – Kim 1957: 24; Townes et al. 1965: 257.

##### Material examined.

***Holotype.*** Korea • 1♂; Shakoji [= currently Sukwangsa at Chuncheon-si, Gangwon-do]; 23. Jul. 1922; T. Uchida leg.; SEHU. ***Non-type material*.** Taiwan • 1♀; Hualien County, Xiulin Township, Cross Island Rd. 113 K, ca 24.188294 N, 121.333768 E (DD); alt. ca 2380 m; 26. Aug. 1989; Light Trap; J. T. Chao leg.; TFRI 00073659 • 1♀; Chiayi County, Alishan Township, Mt. Alishan; alt. 2400 m; 5–9. Aug. 1989; Malaise Trap; L. Y. Chou & S. C. Lin; TARI (measure01) • 1♂; ibid; 5. Aug. 1931; T. Shiraki; TARI (measure07) • 1♀; Nantou County, Ren’ai Township, Meifeng; alt. 2130 m; 23. Sep.–2. Oct. 1979; Malaise Trap; unknown collector; TARI (measure02) • 1♀; ibid; 22–29. Aug. 1979; TARI (measure03) • 1♂; ibid; 18–23. Sep. 1979; TARI (measure08) • 1♀; ibid; Jul. 1984; alt. 2150 m; K. S. Lin & K. C. Chou leg.; TARI (measure04) • 1♀1♂; ibid; Aug. 1984; TARI (measure05) • 3♂♂; Nantou County, Ren’ai Township, Tsuifeng; alt. 2300 m; Aug. 1984; Malaise Trap; K. S. Lin & K. C. Chou leg.; TARI (measure10) • 2♂♂; ibid; Sep. 1984; TARI (measure09) • 1♀; ibid; Oct. 1985; K. S. Lin leg.; TARI (measure06) • 1♀; Nantou County, Xinyi Township, Tungpu; alt. 1200 m; Nov. 1985; K. S. Lin leg.; TARI • 1♀; Nantou County, Ren’ai Township, Shuikuan Road; 29–30. Aug. 2009; Light Trap (UV); H. H. Liang leg.; NMNS ENT 6214-397.

##### Diagnosis.

This species can be distinguished from congeners by the combination of the following characters: fore wing length usually longer than 10.0 mm; POL/OOL = 0.5–0.7; fore wing areolet triangular with stalk, receiving 2m-cu at distal corner (Fig. 10A); fore wing 1cu-a slightly inclivous, distad or almost opposite to M&RS (Fig. 10A); lateromedian longitudinal carinae of propodeum complete and distinct, almost parallel (Fig. 3E); T1 2.7–3.3 × its apical width; gonostyle truncate apically (Fig. 11B); mesosoma black with scutellum reddish-brown; metasoma and legs reddish-brown except hind tarsi yellow (Fig. 3A).

This species is the only one in *Alexeter* with such large body size, black mesosoma, reddish-brown scutellum, and almost entirely reddish-brown legs and metasoma. The Asian species *A. daisetsusanus* Uchida, 1930, *A. dorogawaensis* Uchida, 1934, *A. segmentarius* (Fabricius, 1787), and *A. fallax* (Holmgren, 1857) share a general color pattern of black mesosoma and reddish-brown metasoma. However, the metasomas of these other species are black on at least the posterior tergites, whereas in *A. shakojiensis*, the posterior tergites of the metasoma are reddish-brown.

##### Redescription based on Taiwanese specimens.

The measurements were based on Taiwanese specimens (8 females and 4 males).

**Female.** Head (Fig. 3A–C): matt and granulate, HW/HL = 1.8–2.1 (1.9 ± 0.12); ocelli normal, OD = 0.2–0.3 (0.2 ± 0.03) mm, POL/OD = 0.5–0.9 (0.7 ± 0.13), OOL/OD = 1.0–1.4 (1.2 ± 0.14), POL/OOL = 0.5–0.7 (0.6 ± 0.08); face granulate with strong punctures, FW/FH = 1.5–1.8 (1.7 ± 0.12); clypeus smooth with subventral transverse ridges, truncate on ventral margin, CLW/CLH = 2.6–3.1 (2.9 ± 0.14); MSL/BMW = 0.4–0.6 (0.5 ± 0.07); mandible minutely coriaceous with strong punctures, teeth equal in length; flagellum with 44–51 segments; average ratio of basal five flagellomeres length 2.4: 1.3: 1.2: 1.1: 1.0.

Mesosoma (Fig. 3A, D, E): matt and granulate with dense punctures, mesopleuron with weak rugae; pronotum with epomia strong, carinate at dorso-anterior corner; mesoscutum with MSSL/MSSW = 1.3–1.4 (1.3 ± 0.03), notauli short, distinct near anterior margin; scutellum with SCL/SCW = 1.0–1.3 (1.1 ± 0.14), lateral carina absent; epicnemial carina strong, ~0.8 × height of mesopleuron with short carinae posteriorly; metapleuron with pleural carina and submetapleural carina complete; juxtacoxal carina present posteriorly; propodeum with spiracle circular to suboval, maximum axis 1.0–1.3 (1.0 ± 0.09) × as minimum axis; anterior transverse carina absent or vestigial medially; posterior transverse carina present medially at posterior ~0.4; lateromedian longitudinal carina present, with area petiolaris opened anteriorly; lateral longitudinal carina vestigial; average ratio of hind tarsomere length 4.5: 2.3: 1.8: 1.0: 1.2.

Wings (Fig. 10A): fore wing length 9.7–11.9 (10.9 ± 0.85) mm; areolet open and triangular with stalk 0.4–0.6 (0.5 ± 0.07) × as long as 2rs-m, receiving 2m-cu at distal corner; RMI = 0.6–0.9 (0.7 ± 0.08); 1cu-a slightly inclivous, distad or almost opposite to M&RS, with BNI = 0.1–0.3 (0.2 ± 0.05). Hind wing length 7.0–9.0 (8.2 ± 0.75) mm; NI = 1.3–2.1 (1.7 ± 0.33); distal hamuli 6–9.

Metasoma (Fig. 3A, F–H): matt and minutely coriaceous; T1 2.7–3.3 (3.1 ± 0.19) × as long as posterior width, 7.2–8.7 (7.8 ± 0.55) × as long as anterior width, 1.4–1.5 (1.5 ± 0.06) × as long as length of T2; T1 with latero-median carinae absent, dorso-lateral carina present anteriorly, ventro-lateral carina complete, spiracle at around middle of T1, glymma distinct; T2 1.2–1.5 (1.3 ± 0.11) × as long as posterior width, 1.7–2.4 (1.9 ± 0.21) × as long as anterior width, gastrocoeli indistinct, thyridia semi-circular; ovipositor sheath 3.3–6.2 (4.4 ± 0.98) × as long as its maximum width in lateral view, shorter than apical depth of metasoma.

Color (Fig. 3, 10A): head and mesosoma generally black, except antenna and ventral 0.6 of face gradient from reddish-brown to yellow; clypeus, malar space, and mandibles except teeth yellow; palpi, tegula, scutellum, and postscutellum reddish-brown. Metasoma and legs generally reddish-brown, except glymma and anterior 0.5–0.8 of T1 sometimes black, mid coxa sometimes tinged with black, hind tarsus yellow. Wings hyaline tinged with yellowish-brown, veins and pterostigma reddish-brown.

**Male.** General structure and color similar to female, except mesoscutum with lateral-anterior corner having yellowish- or reddish-brown marking. Male genitalia with gonostyle truncate apically and weakly concave on ventral margin, S9 weakly concave on posterior margin, completely sclerotized (Fig. 11A–C).

HW/HL = 1.7–2.1 (1.9 ± 0.17); OD = 0.22–0.26 (0.24 ± 0.02) mm, POL/OD = 0.6–0.7 (0.6 ± 0.04), OOL/OD = 1.0–1.2 (1.1 ± 0.07), POL/OOL = 0.5–0.7 (0.6 ± 0.08); FW/FH = 1.6–1.8 (1.7 ± 0.10), CLW/CLH = 2.9–3.2 (3.1 ± 0.14), MSL/BMW = 0.4–0.5 (0.5±0.06); flagellum with 46–51 segments; average ratio of basal five flagellomeres length 2.1: 1.3: 1.1: 1.1: 1.0; MSSL/MSSW = 1.3–1.5 (1.4 ± 0.06); SCL/SCW = 0.9–1.2 (1 ± 0.16); maximum axis of propodeal spiracles 1.0–1.1 (1.1 ± 0.06) × as minimum axis; average ratio of hind tarsomere length 4.6: 2.3: 1.8: 1.0: 1.2; fore wing length 10.0–11.0 (10.4 ± 0.49) mm; areolet with stalk 0.4–0.8 (0.6 ± 0.14) × as long as 2rs-m; RMI = 0.7–0.8 (0.7 ± 0.04); BNI = 0.1–0.3 (0.2 ± 0.11); hind wing length 7.1–7.8 (7.5 ± 0.32) mm; NI = 1.4–2.2 (1.8 ± 0.34); distal hamuli 7–10; T1 2.8–3.7 (3.3 ± 0.39) × as long as posterior width, 7.3–8.1 (7.7 ± 0.31) × as long as anterior width, 1.4–1.6 (1.5 ± 0.09) × as long as length of T2; T2 1.3–1.6 (1.4 ± 0.11) × as long as posterior width, 1.9–2.4 (2.1 ± 0.19) × as long as anterior width.

##### Bionomics.

This species has been collected in mountainous areas in Taiwan above 2000 m by Malaise trap or light trap. Hosts are unknown.

##### Distribution.

Korea, China, and Taiwan (new record: Hualien, Nantou, and Chiayi).

##### Remarks.

This is the first record of this species from Taiwan. The amplification of *COI* sequences in this species failed in this study.

#### ﻿ Alexeter flavomaculatus

Taxon classificationAnimaliaHymenopteraIchneumonidae

Chen, Huang & Shiao sp. nov.

4AF2797C-5ED4-5B86-89B2-3066B3088AD2

https://zoobank.org/FBE1DE3A-10D5-40DB-9B37-93DD3F1E43D8

**Figure 4.**
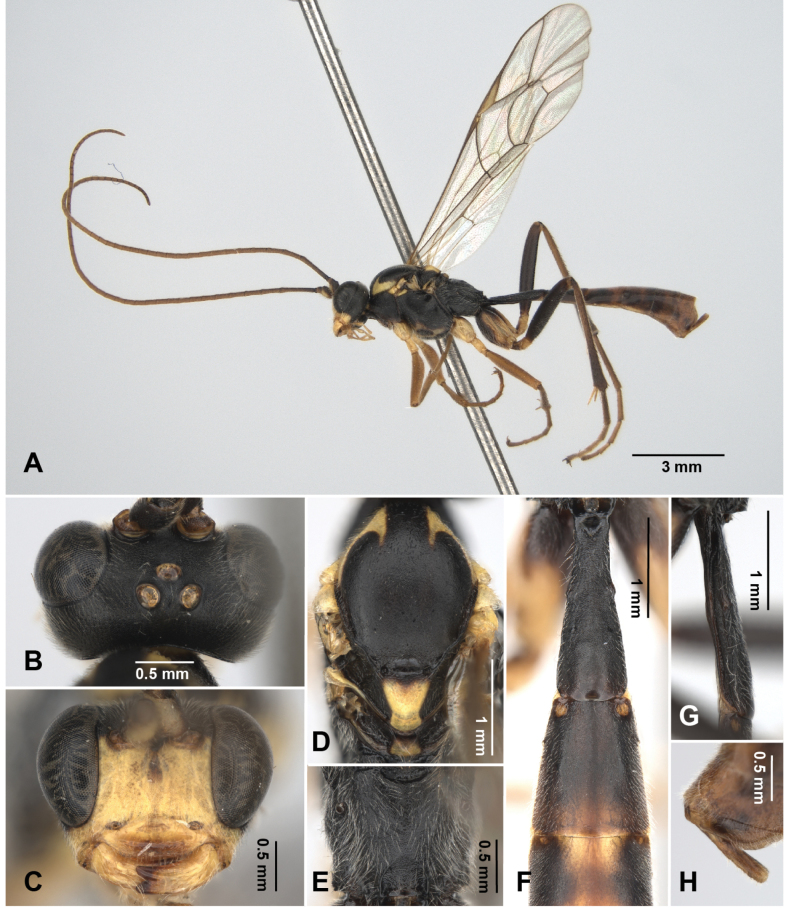
*Alexeter flavomaculatus* sp. nov., holotype, female (TARI). **A.** Lateral habitus; **B.** Head in dorsal view; **C.** Head in anterior view; **D.** Mesoscutum in dorsal view; **E.** Propodeum in dorsal view; **F.** Metasomal tergites 1 and 2 in dorsal view; **G.** Metasomal tergite 1 in lateral view; **H.** Ovipositor.

Figs 4A–H, 10B, 11D–F, 12A–C; Suppl. material 3 Chinese vernacular name: 黃紋亞力姬蜂

##### Material examined.

***Holotype*.** Taiwan • 1♀; Nantou County, Ren’ai Township, Meifeng; alt. 2150 m; May. 1984; Malaise Trap; K. S. Lin & K. C. Chou leg.; TARI (MesoleiYM-F01). ***Paratypes*.** Taiwan • 1♂; ibid; 7–9. May. 1984; TARI MesoleiYM-M01 • 1♂; ibid; Apr. 1984; TARI (MesoleiYM-M02) • 1♂; Nantou County, Ren’ai Township, Sanjiaofeng trail; alt. 2200–2300 m; 11. May. 2024; Sweeping; J. Y. Fan leg.; GenBank: PV223412 (*COI*); NMNS ENT 8951-8 (Mesolei15).

##### Diagnosis.

This species can be distinguished from congeners by the combination of the following characters: smaller ocelli (OD = 0.15–0.19 mm; OOL/OD = 1.2–1.6); fore wing areolet trapezoid with stalk, receiving 2m-cu at distal corner (Fig. 10B); fore wing 1cu-a almost vertical, opposite or slightly distad to M&RS (Fig. 10B); lateromedian longitudinal carinae of propodeum present posteriorly with the area petiolaris opened (Fig. 3E); T1 2.7–3.5 × its posterior width; S9 with subtriangular median area weakly sclerotized (Fig. 11D); mesosoma and metasoma generally black, with latero-anterior corners of mesoscutum, ventral subtriangular marking of mesopleuron (enclosing black area), and median markings on tergites behind T2 yellow (Figs 4A, D–F, 12A–C).

This new species is similar to the Asian species *A. flaviventris* Li & Sun, 2022, *A. albimaculatus* Sheng, Sun & Li, 2020, and *A. zangicus* Sheng, Sun & Li, 2020 which share black bodies and pale markings on the latero-anterior corners of mesoscutum.

It can be distinguished from *A. flaviventris* by the following characters: lateromedian longitudinal carinae of propodeum absent on the anterior and median portions (Fig. 4E) (present and combined as single carina medially in *A. flaviventris*); metasomal tergites and ventral portion of mesopleuron with yellow markings (Figs 4A, F, 12B) (without in *A. flaviventris*).

It can also be distinguished from *A. albimaculatus* by the following characters: face and mandibles yellow (Figs 4C, 12A) (face white and mandibles entirely black in *A. albimaculatus*); fore and mid legs each with yellow coxae and reddish-brown other part(Fig. 4A) (generally black with white tibiae in *A. albimaculatus*); latero-anterior corners of mesoscutum, ventral portion of mesopleuron, and metasomal tergites with yellow markings (Figs 4A, D, F, 12B, C) (marking on mesoscutal corners white, other markings absent in *A. albimaculatus*); antenna without white band (Fig. 4A) (with white band in *A. albimaculatus*); and lateromedian longitudinal carinae of propodeum absent on the anterior and median portions (Fig. 4E) (present in *A. albimaculatus*).

Lastly, it can be distinguished from *A. zangicus* by the following characters: areolet present (Fig. 10B) (absent in *A. zangicus*); face and clypeus yellow (Figs 4C, 12A) (face black and clypeus yellow in *A. zangicus*); truncate ventral margin of clypeus (Figs 4C, 12A) (concave in *A. zangicus*); metasomal tergites black with yellow markings (Fig. 4F) (generally reddish-brown in *A. zangicus*).

##### Description.

The measurements were based on Taiwanese specimens (1 female and 3 males).

**Female** (holotype). Head (Figs 4A–C, 12A): matt and granulate, HW/HL = 1.8; ocelli normal, with OD = 0.2 mm, POL/OD = 0.8, OOL/OD = 1.4, POL/OOL = 0.6; face matt and granulate, FW/FH = 1.6; clypeus polished and smooth with sparse punctures and subventral median elevation, rounded on ventral margin, CLW/CLH = 2.6; MSL/BMW = 0.5; mandible minutely coriaceous with minute punctures, teeth equal in length; flagellum with 45 segments; average ratio of basal five flagellomeres length 2.5: 1.4: 1.1: 1.2: 1.0.

Mesosoma (Figs 4A, D, E, 12B, C): matt and granulate; pronotum with epomia weak, rugose at dorso-anterior corner; mesoscutum with MSSL/MSSW = 1.2, notauli short and indistinct, present near anterior margin; scutellum with SCL/SCW = 1.3, lateral carina absent; epicnemial carina weak, ~0.7 × height of mesopleuron; metapleuron with pleural carina and submetapleural carina strong and complete; juxtacoxal carina present posteriorly; propodeum with spiracle circular, maximum axis 1.0 × as minimum axis; anterior and posterior transverse carinae and lateral longitudinal carina absent; lateromedian longitudinal carina absent on anterior and median portions, present on posterior ~0.3 with area petiolaris opened anteriorly; average ratio of hind tarsomere length 4.0: 2.0: 1.5: 1.0: 1.0.

Wings (Fig. 10B): fore wing length 8.3 mm; areolet open and trapezoid with stalk 0.5 as long as 2rs-m, receiving 2m-cu at distal corner; RMI = 0.7; 1cu-a almost vertical, opposite or slightly distad to M&RS, with BNI = 0.2. Hind wing length 6.0 mm; NI = 2.2; distal hamuli 6–7.

Metasoma (Fig. 4F–H): matt and granulate, except tergites behind T2 weakly coriaceous; T1 3.5 × as long as posterior width, 6.6 × as long as anterior width, 2.3 × as long as length of T2; T1 with latero-median carina absent, dorso-lateral carina and ventro-lateral carina strong and complete, spiracle at around middle of T1, glymma distinct; T2 0.8 × as long as posterior width, 1.3 × as long as anterior width, gastrocoeli shallow and indistinct, thyridia almost circular; ovipositor sheath 6.0 × as long as its maximum width in lateral view, as long as apical depth of metasoma.

Color (Figs 4, 10B): head, mesosoma, and metasoma generally black, and legs generally reddish-brown, except face, dorsal 1/2 of clypeus, malar space, mandibles, palpi, dorso-posterior corner of pronotum, latero-anterior corners of mesoscutum, dorso-anterior corner and ventral subtriangular marking (except enclosing area) of mesopleuron, tegula, scutellum, postscutellum, fore and mid coxae and trochanters, dorsal surface of hind coxa, hind second trochanter, median markings on tergites behind T2 yellow; antenna, marking on ventral 1/2 of clypeus, tinged color of tergites behind T2, ovipositor sheath reddish-brown; ventral side of hind coxa, hind trochanter, and hind femur black. Wings hyaline, veins blackish-brown, and pterostigma reddish-brown.

**Male.** General structure and color similar to female, except notauli long and distinct, lateromedian longitudinal carinae of propodeum vestigial on median portion, and clypeus completely yellow, subtriangular marking with enclosing black area on ventral mesopleuron more distinct (Fig. 11A–C). Male genitalia with gonostyle tapered and rounded apically, S9 weakly concave on posterior margin, with subtriangular median area weakly sclerotized (Fig. 11D–F).

HW/HL = 1.8–1.9 (1.8 ± 0.06); OD = 0.15–0.19 (0.17 ± 0.02) mm, POL/OD = 0.5–0.9 (0.7 ± 0.2), OOL/OD = 1.2–1.6 (1.4 ± 0.18), POL/OOL = 0.4–0.6 (0.5 ± 0.09); FW/FH = 1.4–1.8 (1.6 ± 0.17), CLW/CLH = 3.0–4.0 (3.6 ± 0.54), MSL/BMW = 0.52–0.54 (0.52 ± 0.01); flagellum with 43–45 segments; average ratio of basal five flagellomeres length 2.3: 1.3: 1.2: 1.1: 1.0; MSSL/MSSW = 1.2–1.3 (1.3 ± 0.04); SCL/SCW = 1.1–1.2 (1.1 ± 0.07); maximum axis of propodeal spiracles 1.0–1.2 (1.1 ± 0.1) × as minimum axis; average ratio of hind tarsomere length 4.1: 2.2: 1.8: 1.0: 1.2; fore wing length 7.2–7.8 (7.5 ± 0.29) mm; areolet with stalk 0.4–0.6 (0.5 ± 0.1) × as long as 2rs-m; RMI = 0.7–0.8 (0.8 ± 0.04); BNI = 0.1–0.2 (0.2 ± 0.03); hind wing length 5.2–5.6 (5.4 ± 0.23) mm; NI = 2.0–2.7 (2.4 ± 0.36); distal hamuli 6–7; T1 2.7–3.1 (2.9 ± 0.16) × as long as posterior width, 6.1–6.6 (6.3 ± 0.29) × as long as anterior width, 1.3–1.6 (1.5 ± 0.11) × as long as length of T2; T2 1.2–1.4 (1.2 ± 0.1) × as long as posterior width, 1.6–1.9 (1.7 ± 0.13) × as long as anterior width.

##### Bionomics.

This species has been collected from mountainous areas in Taiwan above 2000 m by Malaise trap or sweeping. Hosts are unknown.

##### Distribution.

Taiwan (Nantou).

##### Etymology.

The specific name *flavomaculatus* is derived from the Latin words *flavo*- (yellow) and *maculatus* (stained or spotted), referring to the yellow markings in the body color pattern of this new species. The name is an adjective.

##### Remarks.

This new species is assigned to the genus *Alexeter* based on both morphological characters and molecular phylogeny. It is nested within *Alexeter* Clade I in the current *COI*-based phylogeny (Fig. 2). Although the notauli are occasionally indistinct in some individuals (whereas they are typically distinct in *Alexeter*), other characters align with the generic definition of *Alexeter*.

#### ﻿ Alexeter hsiaoae

Taxon classificationAnimaliaHymenopteraIchneumonidae

Chen, Huang & Shiao sp. nov.

FD6321A6-7A0C-581B-B8A6-402A1ABEE64D

https://zoobank.org/1A4DE443-FE75-41E4-8A0B-6CDACF674270

**Figure 5.**
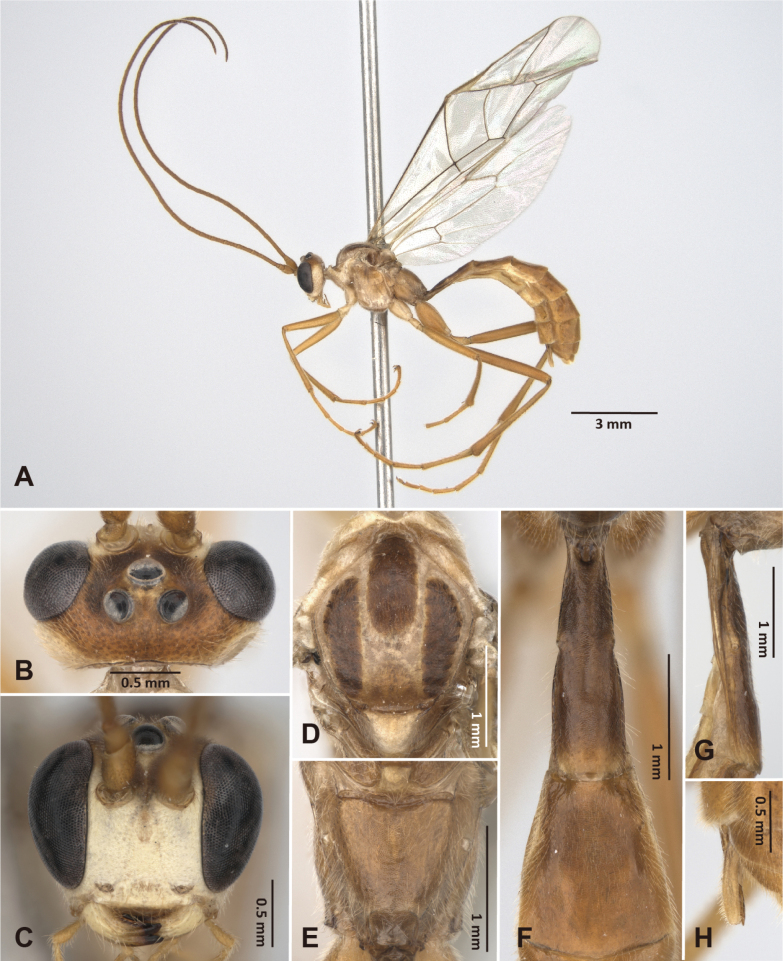
*Alexeter hsiaoae* sp. nov., holotype, female (NMNS ENT 8951-1; Mesolei01). **A.** Lateral habitus; **B.** Head in dorsal view; **C.** Head in anterior view; **D.** Mesoscutum in dorsal view; **E.** Propodeum in dorsal view; **F.** Metasomal tergites 1 and 2 in dorsal view; **G.** Metasomal tergite 1 in lateral view; **H.** Ovipositor.

Figs 5A–H, 10C, 11G–I; Suppl. material 3 Chinese vernacular name: 郁薇亞力姬蜂

##### Material examined.

***Holotype.*** Taiwan • 1♀; Taichung City, Heping District, Mt. Nanhu U-shape valley, 24.369516°N, 121.443366°E (DD); alt. 3450 m; 16. Jul. 2021; Light Trap; C.-L. Huang & Y.-W. Hsiao leg.; GenBank: PV223403 (*COI*); NMNS ENT 8951-1 (Mesolei01). ***Paratypes.*** Taiwan • 1♀4♂♂; same as holotype; GenBank: PV223405, PV223406 (*COI*); NMNS ENT 8951-2–3 (Mesolei02–03), TARI (Mesolei12–13), KPMNH (Mesolei14) • 1♂; Taichung City, Heping District, Eastern Peak of Mt. Syueshan–369 Cabin; alt. 3000–3200 m; 26–27. Aug. 2021; Sweeping; C.-T. Hsu leg.; GenBank: PV223404 (*COI*); NMNS ENT 8951-4 (Mesolei06) • 1♀2♂♂; Hualien County, Wanrung Township, Mt. Antungchunshan, No. C2; 4. Aug. 1993; S. S. Lu leg.; TFRI 00073669–00073671.

##### Diagnosis.

This species can be distinguished from congeners by the combination of the following characters: fore wing length usually shorter than 10.0 mm (7.8–10.4 mm); ocelli large (OD = 0.18–0.25 mm; OOL/OD = 0.6–0.9); POL/OOL = 0.8–1.1; female with flagellum segments 46; fore wing areolet trapezoid with stalk, receiving 2m-cu at distal corner (Fig. 10C); fore wing 1cu-a almost vertical, opposite or slightly distad to M&RS (Fig. 10C); lateromedian longitudinal carinae of propodeum present posteriorly with the area petiolaris opened (Fig. 5E); T1 3.1–4.3 × its posterior width; head generally yellowish-white with frons and vertex brown to blackish-brown (Fig. 5A, B); mesosoma, legs, and metasoma generally yellowish-brown, with mesoscutum having three distinct blackish-brown longitudinal stripes on the lobes (Fig. 5A, D).

This new species is most similar to *A. clavator* (Müller, 1776), *A. monticola* sp. nov., and *A. mediolobus* sp. nov. which share generally yellowish color in head, mesosoma, and metasoma, but can be distinguished from them by the POL/OOL ratio (0.8–1.1 vs 1.3 in Chinese *A. clavator* (Li and Sun 2022) and 1.1–1.5 in *A. mediolobus* sp. nov.), female OOL/OD ratio (0.7–0.9 vs 0.5–0.7 in *A. mediolobus* sp. nov. and *A. monticola* sp. nov.), length-to-posterior-width ratio of T1 (3.1–4.3 vs 2.5–3.1 in *A. mediolobus* sp. nov.), female flagellomere counts (46 vs 40–43 in *A. mediolobus* sp. nov.), color patterns of mesoscutum (three stripes on each lobe vs absent in *A. clavator* and one on the median lobe in *A. mediolobus* sp. nov.), gena (yellowish-white vs yellowish-brown to brown in other species), vertex (brown vs yellowish-brown or orange in *A. monticola* sp. nov.), and fore and mid coxae (yellowish-white vs yellowish-brown in *A. monticola* sp. nov.).

For other yellowish- or reddish-brown species *A. nebulator* (Thunberg, 1822), *A. gracilentus* (Holmgren, 1857), and *A. luteifrons* (Cresson, 1868), this new species can be easily distinguished from them by having a yellowish-white head (black in these species) and three blackish-brown stripes on the lobes of mesoscutum (stripes absent in these species). A comparative table of the measurements, ratios, and colors of this new species and other yellowish- or reddish-brown *Alexeter* species are provided in Suppl. material 3.

Additionally, this new species may also be confused with two species of the genus *Barytarbes*, *B. fulvus* Sheng & Schönitzer, 2008 and *B. nigrimaculatus* Sheng & Sun, 2017, which share a generally yellowish-brown body color and the absence of lateromedian longitudinal carinae on the anterior and median portions of propodeum. However, this new species can be distinguished from both *Barytarbes* species by having mandibular teeth equal in length (Fig. 5C) (lower tooth longer in both species), T1 with distinct glymma (Fig. 5G) (glymma absent in *B. fulvus* and indistinct in *B. nigrimaculatus*), T1 3.1–4.3 × its posterior width (~1.9 × in *B. nigrimaculatus*, 4.0 × in *B. fulvus*), mesoscutum with three distinct blackish-brown longitudinal stripes (Fig. 5D) (without stripes on mesoscutum in *B. fulvus*), and metasomal tergites without black markings (Fig. 5A) (with black markings in *B. nigrimaculatus*).

##### Description.

The measurements were based on Taiwanese specimens (3 females and 7 males).

**Female.** Head (Fig. 5A–C): matt and minutely coriaceous, HW/HL = 1.6–1.9 (1.9, 1.8 ± 0.15); ocelli large, with OD = 0.20–0.25 (0.21, 0.22 ± 0.03) mm, POL/OD = 0.5–0.9 (0.9, 0.7 ± 0.19), OOL/OD = 0.7–0.9 (0.8, 0.8 ± 0.11), POL/OOL = 0.8–1.1 (1.1, 0.9 ± 0.18); face matt and minutely coriaceous, FW/FH = 1.37–1.44 (1.4, 1.41 ± 0.05); clypeus flat, polished and smooth with sparce punctures, truncate on ventral margin, CLW/CLH = 2.9–3.3 (3.3, 3.1 ± 0.28); MSL/BMW = 0.3–0.7 (0.3, 0.5 ± 0.21); mandible smooth with sparce punctures, teeth equal in length; flagellum with 46 (46) segments; average ratio of basal five flagellomeres length 2.5: 1.3: 1.1: 1.1: 1.0.

Mesosoma (Fig. 5A, D, E): matt and minutely coriaceous; pronotum with epomia weak; mesoscutum with MSSL/MSSW = 1.1–1.2 (1.1, 1.1 ± 0.07), notauli short and distinct near anterior margin; scutellum with SCL/SCW = 1.2–1.3 (1.2, 1.3 ± 0.01), lateral carina present anteriorly; epicnemial carina strong, ~0.6 × height of mesopleuron; metapleuron with pleural carina and submetapleural carina complete; juxtacoxal carina absent; propodeum with spiracle circular, maximum axis 1.0–1.1 (1.0, 1.04 ± 0.06) × as minimum axis; anterior transverse carinae absent; posterior transverse carina absent but vestigial medially; lateromedian longitudinal carinae absent on anterior and median portions, present on posterior ~0.3–0.4 with area petiolaris opened anteriorly; lateral longitudinal carinae vestigial posteriorly; average ratio of hind tarsomere length 4.1: 2.2: 1.7: 1.0: 1.2.

Wings (Fig. 10C): fore wing length 8.7–10.4 (8.94, 9.4 ± 0.91) mm; areolet open and trapezoid with stalk 0.4–0.5 (0.4, 0.4 ± 0.05) as long as 2rs-m, receiving 2m-cu at distal corner; RMI = 0.5–0.8 (0.8, 0.7 ± 0.12); 1cu-a almost vertical, opposite or slightly distad to M&RS, with BNI = 0.1–0.2 (0.2, 0.2 ± 0.02). Hind wing length 6.8–7.9 (6.77, 7.2 ± 0.62) mm; NI = 1.7–3 (1.7, 2.2 ± 0.71); distal hamuli 5–6 (6 in left and 5 in right wing).

Metasoma (Fig. 5F–H): matt and minutely coriaceous; T1 3.1–3.8 (3.1, 3.3 ± 0.43) × as long as posterior width, 6.2–8.2 (6.2, 7.0 ± 1.07) × as long as anterior width, 1.3–1.5 (1.3, 1.4±0.09) × as long as length of T2; T1 with latero-median carina absent, dorso-lateral carina present basally, ventro-lateral carina complete, spiracle at around middle of T1, glymma distinct; T2 1.1–1.4 (1.2, 1.2 ± 0.13) × as long as posterior width, 1.9–2.3 (2.1, 2.1 ± 0.2) × as long as anterior width, gastrocoeli indistinct, thyridia deep and semi-circular; ovipositor sheath 3.3–5.6 (3.3, 4.6 ± 1.21) × as long as its maximum width in lateral view, shorter than apical depth of metasoma.

Color (Figs 5, 10C): head yellowish-white, except antenna yellowish-brown; vertex brown; frons and areas between lateral ocelli and eyes blackish-brown. Mesosoma, legs, and metasoma generally yellowish-brown, except pronotum, ventral and anterior area of mesopleuron, mesoscutum, scutellum, fore and mid coxae, and trochanters yellowish-white, three longitudinal stripes on lobes of mesoscutum and T1 dark brown to blackish-brown. Wings hyaline, veins blackish-brown, and pterostigma pale yellowish-brown.

**Male.** General structure and color similar to female. Male genitalia with gonostyle tapered and rounded apically, S9 weakly concave on posterior margin, completely sclerotized (Fig. 11G–I).

HW/HL = 1.7–1.9 (1.8 ± 0.08); OD = 0.18–0.23 (0.21 ± 0.02) mm, POL/OD = 0.6–0.9 (0.7 ± 0.12), OOL/OD = 0.6–0.8 (0.7 ± 0.09), POL/OOL = 0.9–1.1 (1 ± 0.08); FW/FH = 1.3–1.7 (1.4 ± 0.13), CLW/CLH = 3.0–3.6 (3.3 ± 0.29), MSL/BMW = 0.3–0.4 (0.3 ± 0.05); flagellum with 42–47 segments; average ratio of basal five flagellomeres length 2.3: 1.3: 1.1: 1.1: 1.0; MSSL/MSSW = 1.1–1.3 (1.2 ± 0.09); SCL/SCW = 1.0–1.4 (1.2 ± 0.12); maximum axis of propodeal spiracles 1.0–1.2 (1.1 ± 0.09) × as minimum axis; average ratio of hind tarsomere length 3.8: 2.0: 1.6: 1.0: 1.1; fore wing length 7.8–9.7 (8.9 ± 0.63) mm; areolet with stalk 0.3–0.5 (0.4 ± 0.06) × as long as 2rs-m; RMI = 0.6–0.8 (0.7 ± 0.09); BNI = 0.1–0.2 (0.2 ± 0.03); hind wing length 5.7–7.0 (6.4 ± 0.47) mm; NI = 1.8–3.0 (2.5 ± 0.43); distal hamuli 4–7; T1 3.5–4.3 (3.9 ± 0.29) × as long as posterior width, 7.0–10.4 (8.2 ± 1.18) × as long as anterior width, 1.3–1.5 (1.4 ± 0.06) × as long as length of T2; T2 1.2–1.6 (1.4 ± 0.16) × as long as posterior width, 1.6–2.6 (2.2 ± 0.32) × as long as anterior width.

##### Bionomics.

This species has been collected from high-elevation mountains in Taiwan above 3000 m by light trap or sweeping. Hosts are unknown.

##### Distribution.

Taiwan (Hualien andTaichung).

##### Etymology.

The eponym of the specific name *hsiaoae* is the Chinese family name ‘Hsiao’ combined with the feminine suffix ‘-*ae*’. It is dedicated to memory the deceased collector of the type series, Ms. Yu-Wei Hsiao. The name is a noun in the genitive case.

##### Remarks.

Large variation in the MSL/BMW ratio is observed in this species, ranges from 0.3 to 0.7. Despite variation in structures, the color patterns of the gena, coxae, and mesoscutum are consistent and can serve as crucial diagnostic characters. *Alexeter hsiaoae* sp. nov. is nested within *Alexeter* Clade I in the current *COI*-based phylogeny (Fig. 2).

#### ﻿ Alexeter mediolobus

Taxon classificationAnimaliaHymenopteraIchneumonidae

Chen, Huang & Shiao sp. nov.

83E0F047-9309-52A7-8AD3-33EE4828E7EA

https://zoobank.org/09211B57-0E23-42AE-93DC-E39B58DC4444

**Figure 6.**
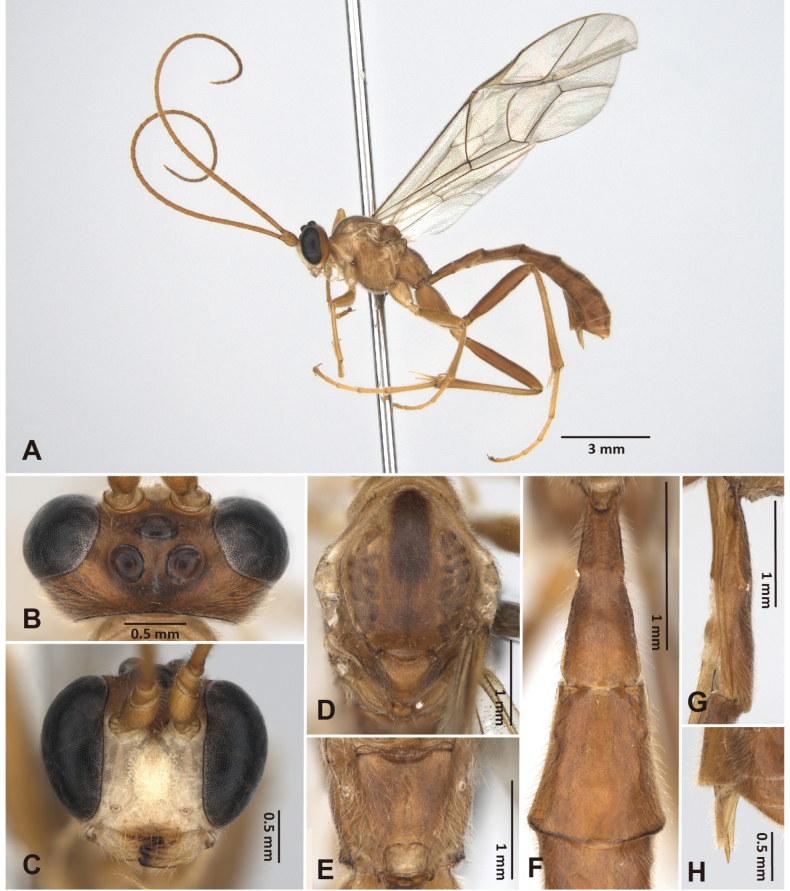
*Alexeter mediolobus* sp. nov., holotype, female (TARI). **A.** Lateral habitus; **B.** Head in dorsal view; **C.** Head in anterior view; **D.** Mesoscutum in dorsal view; **E.** Propodeum in dorsal view; **F.** Metasomal tergites 1 and 2 in dorsal view; **G.** Metasomal tergite 1 in lateral view; **H.** Ovipositor.

Figs 6A–H, 10D, 11J–L; Suppl. material 3 Chinese vernacular name: 中褐亞力姬蜂

##### Material examined.

***Holotype.*** Taiwan • 1♀; Hualien County, Jian Township, Ci’en–Bilu Giant Tree; alt. 2000 m; 27. May. 2020; Light Trap; C. L. Huang & L. H. Wang leg.; GenBank: PV223410 (*COI*); TARI (Mesolei07). ***Paratypes.*** Taiwan • 1♀; Nantou County, Ren’ai Township, Meifeng, Tai-14A highway 14.7 K, 24.092463°N, 121.175501°E (DD); alt. 2180 m; 10. May. 2024; Light Trap; H.-P. Chen leg.; GenBank: PV223411 (*COI*); NMNS ENT 8951-7 (Mesolei16) • 1♀; Nantou County, Ren’ai Township, Meifeng; alt. 2150 m; Jun. 1984; Malaise Trap; K. S. Lin & K. C. Chou leg.; TARI (measure01) • 3♀♀; ibid; May. 1984; TARI (measure02–04) • 1♂; ibid; 22. May. 1984; TARI (measure05) • 1♂; Nantou County, Ren’ai Township, Meifeng; 6–26. Apr. 1997; C.S. Lin & W.T. Yang leg.; NMNS ENT 3028-591.

##### Diagnosis.

This species can be distinguished from congeners by the combination of the following characters: fore wing length usually shorter than 10.0 mm (7.7–10.1 mm); ocelli large (OD = 0.20–0.27 mm; OOL/OD = 0.5–0.7); POL/OOL = 1.1–1.5; female with flagellum segments 40–43; fore wing areolet trapezoid with stalk, receiving 2m-cu at distal corner (Fig. 10D); fore wing 1cu-a almost vertical and opposite to M&RS (Fig. 10D); lateromedian longitudinal carinae of propodeum present posteriorly with the area petiolaris almost closed (Fig. 6E); posterior transverse carina present (Fig. 6E); T1 2.5–3.1 × its posterior width; head, mesosoma, legs, and metasoma generally brown to yellowish-brown, with mesoscutum having single blackish-brown longitudinal stripe on the median lobe (Fig. 6A, D).

This new species is most similar to *A. clavator*, *A. hsiaoae* sp. nov., and *A. monticola* sp. nov. in body color but can be distinguished from them by the POL/OOL ratio (1.1–1.5 vs 0.8–1.1 in *A. hsiaoae* sp. nov. and 0.8–1.2 in *A. monticola* sp. nov.), female OOL/OD ratio (0.5–0.7 vs 0.7–0.9 in *A. hsiaoae* sp. nov.), length-to-posterior-width ratio of T1 (2.5–3.1 vs 3.1–4.3 in *A. hsiaoae* sp. nov., 3.4–3.9 in *A. monticola* sp. nov., and ~3.3 in Chinese *A. clavator*), female flagellomere counts (40–43 vs 46 in *A. hsiaoae* sp. nov. and 46–52 in *A. monticola* sp. nov.), color patterns of mesoscutum (one stripe on the median lobe vs absent in *A. clavator* and three stripes on each lobe in *A. hsiaoae* sp. nov. and *A. monticola* sp. nov.), gena (brown vs yellowish-white in *A. hsiaoae* sp. nov. and sometimes orange in *A. monticola* sp. nov.), and fore and mid coxae (yellowish-brown vs yellowish-white in *A. hsiaoae* sp. nov.).

This new species can also be distinguished from other yellowish- and reddish-brown species *A. nebulator*, *A. gracilentus*, and *A. luteifrons* by having yellowish-brown head (black in these species) and the blackish-brown stripe on the median lobe of mesoscutum (yellowish-brown in these species). A comparative table of the measurements, ratios, and colors of this new species and other yellowish- or reddish-brown *Alexeter* species are provided in Suppl. material 3.

##### Description.

The measurements are based on Taiwanese specimens (6 females and 1 male).

**Female.** Head (Fig. 6A–C): matt and minutely coriaceous, HW/HL = 1.7–2.1 (1.7, 1.9 ± 0.12); ocelli large, with OD = 0.20–0.27 (0.27, 0.25 ± 0.03) mm, POL/OD = 0.6–0.7 (0.6, 0.7 ± 0.05), OOL/OD = 0.5–0.7 (0.5, 0.5 ± 0.06), POL/OOL = 1.1–1.5 (1.1, 1.2 ± 0.13); face matt and minutely coriaceous, FW/FH = 1.4–1.6 (1.5, 1.5 ± 0.08); clypeus matt and evenly punctate with subventral median elevation, truncate or slightly concave on ventral margin, CLW/CLH = 3.0–3.5 (3.5, 3.2 ± 0.19); MSL/BMW = 0.3–0.4 (0.3, 0.3 ± 0.04); mandible densely punctate in dorsal surface, teeth equal in length; flagellum with 40–43 (43) segments; average ratio of basal five flagellomeres length 2.2: 1.2: 1.1: 1.1: 1.0.

Mesosoma (Fig. 6A, D, E): matt and granulate; pronotum with epomia absent, carinate at dorso-anterior corner; mesoscutum with MSSL/MSSW = 1.1–1.2 (1.1, 1.2 ± 0.06), notauli short, distinct near anterior margin; scutellum with SCL/SCW = 1.0–1.4 (1.4, 1.3 ± 0.15), lateral carina present at base; epicnemial carina strong, ~0.7 × height of mesopleuron; metapleuron with pleural carina and submetapleural carina complete; juxtacoxal carina present posteriorly; propodeum with spiracle suboval, maximum axis 1.0–1.2 (1.0, 1.1 ± 0.08) × as minimum axis; anterior transverse carina absent; posterior transverse carina present medially; lateromedian longitudinal carinae absent on anterior and median portions, present on posterior ~0.2 with area petiolaris almost closed; lateral longitudinal carinae weak; average ratio of hind tarsomere length 3.9: 2.1: 1.6: 1.0: 1.2.

Wings (Fig. 10D): fore wing length 7.7–10.1 (10.12, 9.4 ± 0.84) mm; areolet open and trapezoid with stalk 0.3–0.4 (0.3, 0.3 ± 0.05) as long as 2rs-m, receiving 2m-cu at distal corner; RMI = 0.6–0.7 (0.7, 0.6 ± 0.05); 1cu-a almost vertical and opposite to M&RS, with BNI = 0.1–0.2 (0.1, 0.1 ± 0.04). Hind wing length 5.6–7.4 (7.39, 6.8 ± 0.6) mm; NI = 1.9–2.8 (2.4 ± 0.32); distal hamuli 6–8 (6).

Metasoma (Fig. 6F–H): matt and coriaceous; T1 2.5–3.1 (2.8, 2.9 ± 0.22) × as long as posterior width, 5.8–8.0 (7.7, 7.0 ± 0.91) × as long as anterior width, 1.4–1.5 (1.5, 1.5 ± 0.03) × as long as length of T2; T1 with latero-median carina absent, dorso-lateral carina present on anterior 0.1 and posterior 0.2, ventro-lateral carina complete, spiracle at around middle of T1, glymma distinct; T2 0.9–1.3 (1.1, 1.2 ± 0.14) × as long as posterior width, 1.4–2.1 (1.8, 1.8 ± 0.22) × as long as anterior width, gastrocoeli indistinct, thyridia indistinct and circular; ovipositor sheath 2.7–5.5 (3.5, 3.5 ± 1.02) × as long as its maximum width in lateral view, shorter than apical depth of metasoma.

Color (Fig. 6, 10D): head, mesosoma, legs, and metasoma generally brown, except face, dorsal 0.7 of clypeus, malar space, gena, mandible base, and dorso-posterior corner of pronotum yellowish-white; palpi, pronotum, dorso-anterior corner and speculum of mesopleuron, fore and mid coxae, and all trochanters and tarsi pale yellowish-brown; frons, vertex, median lobe of mesoscutum in anterior 1/2, lateral sides of scutellum blackish-brown. Wings hyaline, veins blackish-brown, pterostigma pale yellowish-brown.

**Male.** General structure and color similar to female. Male genitalia with gonostyle tapered and rounded apically, S9 weakly concave on posterior margin, completely sclerotized (Fig. 11J–L).

HW/HL = 1.9; OD = 0.25 mm, POL/OD = 0.6, OOL/OD = 0.5, POL/OOL = 1.3; FW/FH = 1.5, CLW/CLH = 3.0, MSL/BMW = 0.4; flagellum segments broken; average ratio of basal five flagellomeres length 2.2: 1.3: 1.1: 1.2: 1.0; MSSL/MSSW = 1.1; SCL/SCW = 1.2; maximum axis of propodeal spiracles 1.1 × as minimum axis; average ratio of hind tarsomere length 3.7: 2.1: 1.6: 1.0: 1.3; fore wing length 8.9 mm; areolet with stalk 0.1 × as long as 2rs-m; RMI = 0.3; BNI = 0.1; hind wing length 6.6 mm; NI = 1.9; distal hamuli 6; T1 2.9 × as long as posterior width, 8.9 × as long as anterior width, 1.5 × as long as length of T2; T2 1.3 × as long as posterior width, 1.7 × as long as anterior width.

##### Bionomics.

This species has been collected from mountainous areas in Taiwan above 2000 m by Malaise trap or light trap. Hosts are unknown.

##### Distribution.

Taiwan (Hualien and Nantou).

##### Etymology.

The specific name *mediolobus* is derived from the Latin *medio*- (meaning medial) plus *lobus* (meaning lobe). It refers to the median lobe of mesoscutum in this species with a distinct blackish-brown marking. The name is an adjective.

##### Remarks.

In dried specimens of this new species, the lateral lobes of the mesoscutum appear darker than in fresh specimens, which can sometimes lead to misinterpretation as longitudinal stripe patterns. It is nested within *Alexeter* Clade I in the current *COI*-based phylogeny (Fig. 2). Endosymbiont co-amplification was observed in this species when amplifying the *COI*-5P region of the *COI* gene using the LCO1490/HCO2198 primer pair or other primers targeting the same binding site, resulting in noisy chromatograms. Successful amplification of *COI* sequences was achieved using the primer pair C1-J-1718/C1-N-2329 (Rousse et al. 2016).

#### ﻿ Alexeter monticola

Taxon classificationAnimaliaHymenopteraIchneumonidae

Chen, Huang & Shiao sp. nov.

2975436C-3458-5226-ABF2-F6E7E24AD1C0

https://zoobank.org/8C93C002-1524-4CAB-8FEF-17B715830265

**Figure 7.**
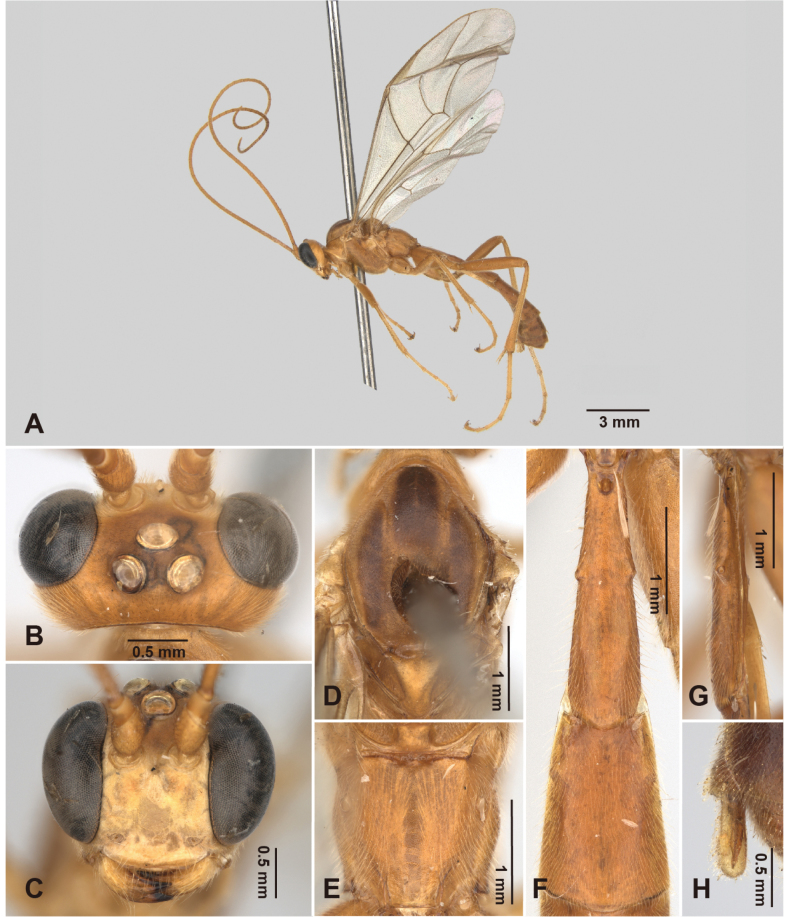
*Alexeter monticola* sp. nov., holotype, female (NMNS ENT 7392-1403). **A.** Lateral habitus; **B.** Head in dorsal view; **C.** Head in anterior view; **D.** Mesoscutum in dorsal view; **E.** Propodeum in dorsal view; **F.** Metasomal tergites 1 and 2 in dorsal view; **G.** Metasomal tergite 1 in lateral view; **H.** Ovipositor.

Figs 7A–H, 10E; Suppl. material 3 Chinese vernacular name: 山亞力姬蜂

##### Material examined.

***Holotype.*** Taiwan • 1♀; Nantou County, Ren’ai Township, Yuanfeng; alt. 2700–2800m; 2. Aug.–8. Sep. 2005; Malaise Trap (KCN); C.S. Lin & W.T. Yang leg.; NMNS ENT 7392-1403. ***Paratypes.*** Taiwan • 1♀; Nantou County, Ren’ai Township, Yuanfeng; alt. 2700–2800m; 13. Jun.–18. Jul. 2001; Malaise Trap (KCN); C.S. Lin & W.T. Yang leg.; NMNS ENT 4229-2746 • 1♀; ibid; 8. Sep.–4. Oct. 2005; NMNS ENT 7393-612 • 1♀; ibid; 9. Jul.–13. Aug. 2002; NMNS ENT 4373-1645 • 2♀♀; ibid; 13. Aug.–10. Sep. 2002; NMNS ENT 4373-491, NMNS ENT 4373-374 • 1♀; ibid; 7. Aug.–11. Sep. 2001; NMNS ENT 4229-98 • 1♀; Nantou County, Ren’ai Township, Tsuifeng; alt. 2300 m; 1–3. Sep. 1982; Malaise Trap; L. Y. Chou & K. C. Chou leg.; TARI (measure01) • 2♀♀; ibid; Sep. 1984; K. S. Lin & K. C. Chou leg.; TARI (measure02–03).

##### Diagnosis.

This species can be distinguished from congeners by the combination of the following characters: fore wing length longer than 10.0 mm (10.7–12.0 mm); ocelli large (OD = 0.21–0.29 mm; OOL/OD = 0.5–0.8); POL/OOL = 0.8–1.2; female with flagellum segments 46–52; fore wing areolet triangular with stalk, receiving 2m-cu at distal corner (Fig. 10E); fore wing 1cu-a vertical and opposite to M&RS (Fig. 10E); lateromedian longitudinal carinae of propodeum present posteriorly and forming the area petiolaris opened (Fig. 7E); posterior transverse carina absent (Fig. 7E); metasomal T1 3.4–3.9 × its posterior width; head, mesosoma, legs, and metasoma generally yellowish-brown to orange, with mesoscutum having three blackish-brown longitudinal stripes on the lobes (Fig. 7A, D).

This new species is most similar to *A. clavator*, *A. hsiaoae* sp. nov., and *A. mediolobus* sp. nov. in body color but can be distinguished by the POL/OOL ratio (0.8–1.2 vs 1.1–1.5 in *A. mediolobus* sp. nov.), female OOL/OD ratio (0.5–0.7 vs 0.7–0.9 in *A. hsiaoae* sp. nov.), length-to-posterior-width ratio of T1 (3.4–3.9 vs 2.5–3.1 in *A. mediolobus* sp. nov., and ~3.3 in Chinese *A. clavator*), female flagellomere counts (46–52 vs 40–43 in *A. mediolobus* sp. nov.), color patterns of mesoscutum (three stripes on each lobe vs absent in *A. clavator* and one stripe on the median lobe in *A. mediolobus* sp. nov.), gena (orange or yellowish-brown vs yellowish-white in *A. hsiaoae* sp. nov.), and fore and mid coxae color (yellowish-brown vs yellowish-white in *A. hsiaoae* sp. nov.).

This new species can also be distinguished from other yellowish- and reddish-brown species *A. nebulator*, *A. gracilentus*, and *A. luteifrons* by having yellowish-brown head (black in these species) and three brown stripes on the lobes of mesoscutum (stripes absent in these species). A comparative table of measurements, ratios, and colors of this new species and other yellowish- or reddish-brown *Alexeter* species are provided in Suppl. material 3.

##### Description.

The measurements are based on Taiwanese specimens (10 females).

**Female.** Head (Fig. 7A–C): matt and minutely coriaceous, HW/HL = 1.6–1.8 (1.8, 1.7 ± 0.08); ocelli large, with OD = 0.21–0.29 (0.24, 0.25 ± 0.03) mm, POL/OD = 0.6–0.8 (0.6, 0.7 ± 0.09), OOL/OD = 0.5–0.7 (0.7, 0.7 ± 0.09), POL/OOL = 0.8–1.2 (0.9, 1.0 ± 0.14); face matt and coriaceous, FW/FH = 1.3–1.6 (1.5, 1.5 ± 0.07); clypeus polished and smooth with sparce punctures and subventral median elevation, truncate on ventral margin, CLW/CLH = 3.0–3.8 (3.4, 3.4 ± 0.27); MSL/BMW = 0.4–0.5 (0.4, 0.4 ± 0.03); mandible smooth with sparce punctures, teeth equal in length; flagellum with 46–52 (49) segments; average ratio of basal five flagellomeres length 2.2: 1.3: 1.1: 1.0: 1.0.

Mesosoma (Fig. 7A, D, E): matt and granulate; pronotum with epomia absent, weakly and densely carinate at dorso-anterior corner; mesoscutum with MSSL/MSSW = 1.1–1.3 (1.3, 1.2 ± 0.06), notauli distinct on anterior ~0.3; scutellum with SCL/SCW = 0.9–1.4 (1.1, 1.2 ± 0.16), lateral carina absent; epicnemial carina strong, ~0.5 × height of mesopleuron; metapleuron with pleural carina and submetapleural carina complete; juxtacoxal carina absent; propodeum with spiracle circular or suboval, maximum axis 1.0–1.1 (1.1, 1.1 ± 0.05) × as minimum axis; anterior and posterior transverse carinae absent; lateromedian longitudinal carinae absent on anterior and median portions, present on posterior ~0.2 with area petiolaris opened anteriorly; lateral longitudinal carinae vestigial posteriorly; average ratio of hind tarsomere length 4.0: 2.0: 1.6: 1.0: 1.1.

Wings (Fig. 10E): fore wing length 10.7–12.0 (10.74, 11.2 ± 0.48) mm; areolet open and triangular with stalk 0.3–0.5 (0.4, 0.4 ± 0.05) as long as 2rs-m, receiving 2m-cu at distal corner; RMI = 0.6–1.0 (0.7, 0.7 ± 0.10); 1cu-a vertical and almost opposite to M&RS, with BNI = 0.1–0.2 (0.1, 0.17 ± 0.03). Hind wing length 8.0–9.0 (8.01, 8.3 ± 0.37) mm; NI = 1.8–3.7 (2.1, 2.3 ± 0.61); distal hamuli 6–9 (6 in left and 7 in right wing).

Metasoma (Fig. 7F–H): matt and granulate; T1 3.4–3.9 (3.9, 3.5 ± 0.16) × as long as posterior width, 6.7–8.2 (7.3, 7.5 ± 0.55) × as long as anterior width, 1.4–1.6 (1.4, 1.4 ± 0.06) × as long as length of T2; T1 with latero-median carina absent, dorso-lateral carina present on basal ~0.1 and apical 0.3, ventro-lateral carina complete, spiracle at around basal 0.4, glymma distinct; T2 1.2–1.5 (1.5, 1.3 ± 0.08) × as long as posterior width, 1.9–2.4 (2.3, 2.1 ± 0.17) × as long as anterior width, gastrocoeli indistinct, thyridia long ellipse; ovipositor sheath 2.3–3.3 (2.8, 2.8 ± 0.26) × as long as its maximum width in lateral view, shorter than apical depth of metasoma.

Color (Fig. 7, 8E): head, mesosoma, legs, and metasoma generally yellowish-brown to orange, except face, clypeus, and malar space yellowish-white; frons (in most paratypes) and three longitudinal stripes on lobes of mesoscutum blackish-brown; metasoma in some paratypes brown. Wings hyaline, veins yellowish-brown, and pterostigma pale yellowish-brown.

**Figure 8.**
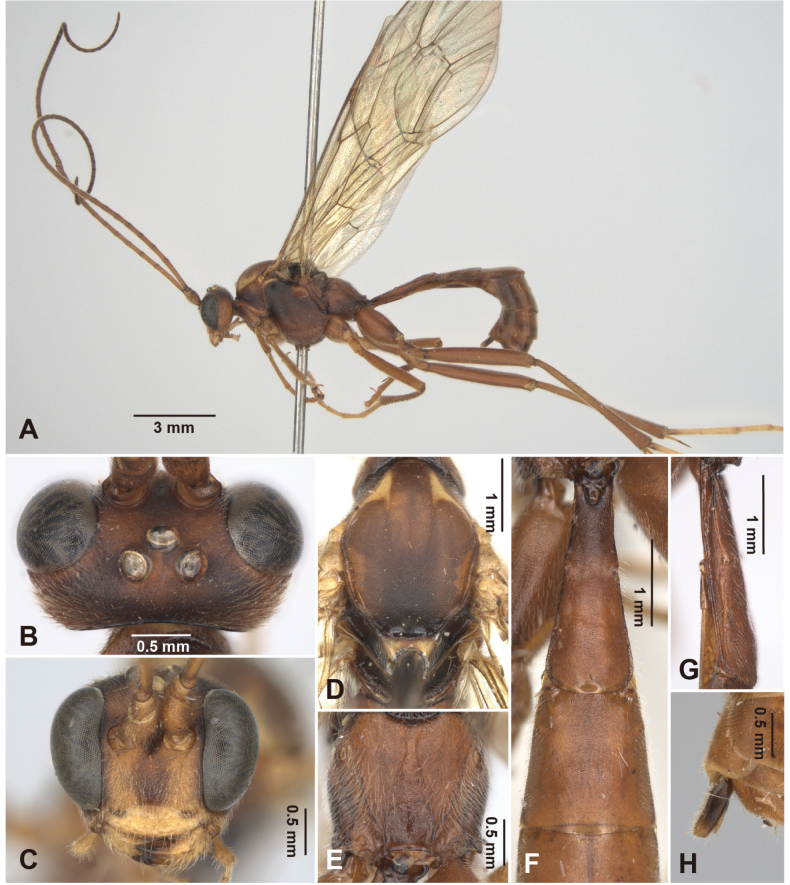
*Alexeter pseudozangicus* sp. nov., holotype, female (TARI). **A.** Lateral habitus; **B.** Head in dorsal view; **C.** Head in anterior view; **D.** Mesoscutum in dorsal view; **E.** Propodeum in dorsal view; **F.** Metasomal tergites 1 and 2 in dorsal view; **G.** Metasomal tergite 1 in lateral view; **H.** Ovipositor.

**Male.** Unknown.

##### Bionomics.

This species has been collected from mountainous areas in Taiwan above 2300 m by Malaise trap. Hosts are unknown.

##### Distribution.

Taiwan (Nantou).

##### Etymology.

The specific name *monticola* is derived from Latin, meaning existing or having a habitat in or on mountains. The name is an adjective.

##### Remarks.

Morphological variations in the color of the frons and metasoma were observed within the type series, with some paratypes exhibiting blackish-brown frons and metasoma. The amplification of *COI* sequences in this species failed in this study.

Despite this new species sharing slightly overlapping OOL/OD ratios, flagellomere counts, and distal hamuli counts with its most similar species, *A. hsiaoae* sp. nov., the diagnostic color patterns (gena, vertex, fore and mid coxae) are clearly distinct between the two species. *A. hsiaoae* sp. nov. exhibits a stable coloration across multiple distant high-mountain localities, including Mt. Nanhu, Mt. Shueshan, and Mt. Antungchunshan. In contrast, *A. monticola* sp. nov. shows minor color variation on the frons within a population collected from a single locality but remains distinguishable based on the aforementioned color patterns. Additionally, their elevational distribution does not overlap among our samples. Therefore, we consider *A. monticola* sp. nov. to be an independent species from *A. hsiaoae* sp. nov.

#### ﻿ Alexeter pseudozangicus

Taxon classificationAnimaliaHymenopteraIchneumonidae

Chen, Huang & Shiao sp. nov.

1EE83A6E-A8A0-58A1-9269-2EB8B22A063B

https://zoobank.org/E5CB94AB-155C-4C99-8DB7-BFA40A578699

Figs 8A–H, 10F, 11P, Q, 12D–F; Suppl. material 3 Chinese vernacular name: 偽藏亞力姬蜂

##### Material examined.

***Holotype.*** Taiwan • 1♀; Nantou County, Ren’ai Township, Tsuifeng; alt. 2300 m; Nov. 1984; Malaise Trap; K. S. Lin & K. C. Chou leg.; TARI (MesileiNA-F01). ***Paratypes.*** Taiwan • 1♀; Nantou County, Ren’ai Township, Tsuifeng; alt. 2300 m; Dec. 1984; TARI (MesileiNA-F02) • 2♀♀; ibid; Oct. 1984; TARI (MesileiNA-F03–04) • 1♀; Nantou county, Ren’ai Township, Yuanfeng; alt. 2700–2800 m; 10. Aug.–9. Sep. 2004; Malaise Trap; C. S. Lin & W. T. Yang leg.; NMNS ENT 6671-1395 • 1♀; ibid; 2. Aug.–8. Sep. 2005; NMNS ENT 7392-1404 • 1♂; Nantou County, Ren’ai Township, Meifeng; alt. 2150 m; 7–9. May. 1984; Malaise Trap; K. S. Lin & S. C. Lin leg.; TARI (MesileiNA-M01).

##### Diagnosis.

This species can be distinguished from congeners by the combination of the following characters: fore wing length usually longer than 10.0 mm (9.6–12.2 mm); ocelli normal (OD = 0.19–0.25 mm; OOL/OD = 0.8–1.1); POL/OOL = 0.7–1.1; clypeus rounded on ventral margin, mandibles with lower tooth slightly longer than upper tooth (Figs 8C, 12D); fore wing areolet absent (Fig. 10F); fore wing 1cu-a inclivous and distad to M&RS (Fig. 10F); lateromedian longitudinal carinae of propodeum complete, with the area petiolaris closed (Fig. 8E); posterior transverse carina present (Fig. 8E); male gonostyle broad (Fig. 11Q); general body color reddish-brown or brown, with yellow markings on the latero-anterior corners of mesoscutum (Figs 8, 12E, F).

This new species is most similar to *A. zangicus* Sheng, Sun & Li, 2020 which shares the absence of fore wing areolet and the yellow markings on the latero-anterior corners of mesoscutum. It can be distinguished from *A. zangicus* by the following characters: body color generally brown to reddish-brown (Figs 8, 12E, F) (black in *A. zangicus*); face yellowish-brown with median blackish-brown marking in female and yellow in male, clypeus entirely yellow (Figs 8C, 12D) (face black and clypeus yellow in *A. zangicus*); rounded ventral margin of clypeus (Figs 8C, 12D) (concave in *A. zangicus*).

It is also similar to the red-type male of *A. rufispeculus* sp. nov. which shares the reddish-brown body color and mesoscutal yellow marking but differs in the following characters: clypeus rounded (Figs 8C, 12D) (truncate in *A. rufispeculus*); mandible with lower tooth slightly longer than upper tooth (Figs 8C, 12D) (equal in length in *A. rufispeculus*); areolet absent (Fig. 10F) (present in *A. rufispeculus*); lateromedian longitudinal carinae of propodeum complete (Fig. 8E) (absent on the anterior and median portions of propodeum in *A. rufispeculus*); T1 2.3–3.0 × its posterior width (3.0–4.3 × in *A. rufispeculus*); and male gonostyle broad (Fig. 11Q) (normal in *A. rufispeculus*).

##### Description.

The measurements are based on Taiwanese specimens (6 females and 1 male).

**Female.** Head (Figs 8A–C, 12D): polished and minutely coriaceous, HW/HL = 1.6–1.8 (1.7, 1.7 ± 0.09); ocelli normal to large, with OD = 0.19–0.25 (0.19, 0.22 ± 0.02) mm, POL/OD = 0.6–1.0 (0.7, 0.8 ± 0.13), OOL/OD = 0.8–1.1 (1.1, 0.9 ± 0.12), POL/OOL = 0.7–1.1 (0.7, 0.9 ± 0.16); face polished and minutely coriaceous, FW/FH = 1.4–1.7 (1.7, 1.5 ± 0.1); clypeus flat, polished and smooth with sparse punctures, rounded on ventral margin, CLW/CLH = 2.8–3.3 (2.8, 3.1 ± 0.23); MSL/BMW = 0.3–0.5 (0.4, 0.4 ± 0.07); mandible smooth with sparce punctures, lower tooth slightly longer than upper tooth; flagellum with 42–48 (48) segments; average ratio of basal five flagellomeres length 2.5: 1.3: 1.2: 1.1: 1.0.

Mesosoma (Figs 8D, E, 12E, F): matt and granulate with dense punctures, mesopleuron minutely coriaceous with weak rugae behind epicnemial carina; pronotum with epomia absent, rugose on dorso-anterior corner; mesoscutum with MSSL/MSSW = 1.2–1.3 (1.2, 1.2 ± 0.03), notauli long, reaching 1/2 of mesoscutum; scutellum with SCL/SCW = 1.0–1.4 (1.4, 1.2 ± 0.16), lateral carina absent; epicnemial carina weak, as long as height of mesopleuron; metapleuron with pleural carina and submetapleural carina complete; juxtacoxal carina long and jointing submetapleural carina anteriorly; propodeum with spiracle suboval, maximum axis 1.1–1.3 (1.1, 1.2 ± 0.11) × as minimum axis; anterior transverse carina absent; posterior transverse carina present medially at posterior ~0.4; lateromedian longitudinal carinae present on anterior and median portions, with area petiolaris closed; lateral longitudinal carinae well-developed; average ratio of hind tarsomere length 4.4: 2.2: 1.6: 1.0: 1.1.

Wings (Fig. 10F): fore wing length 10.1–12.2 (11.99, 11.4 ± 0.77) mm; areolet absent; RMI = 0.7–0.9 (0.9, 0.8 ± 0.11); 1cu-a inclivous and distad to M&RS, with BNI = 0.2–0.3 (0.2, 0.3 ± 0.03). Hind wing length 7.4–8.8 (8.65, 8.4 ± 0.54) mm; NI = 2.3–4.3 (3.0, 3.1 ± 0.75); distal hamuli 7–8 (8).

Metasoma (Fig. 8F–H): polished and minutely coriaceous; T1 2.3–2.8 (2.5, 2.7 ± 0.21) × as long as posterior width, 5.8–7.0 (6.2, 6.4 ± 0.45) × as long as anterior width, 1.5–1.7 (1.5, 1.6 ± 0.09) × as long as length of T2; T1 with latero-median carina absent, dorso-lateral carina weak, distinct anteriorly, ventro-lateral carina complete, spiracle at around middle of T1, glymma distinct; T2 0.9–1.2 (1.0, 1.0 ± 0.08) × as long as posterior width, 1.3–1.5 (1.4, 1.4 ± 0.07) × as long as anterior width, gastrocoeli indistinct, thyridia indistinct and semi-circular; ovipositor sheath 3.1–4.6 (4.6, 3.7 ± 0.59) × as long as its maximum width in lateral view, shorter than apical depth of metasoma.

Color (Figs 8, 10F): head, mesosoma, legs, and metasoma generally brown to reddish-brown, except frons, areas between lateral ocelli and eyes, median area of face, posterior area of mesoscutum, pronotum, dorsal portion of mesopleuron, ventro-apical metapleuron, glymma of T1, and ovipositor sheath blackish-brown or tinged with blackish-brown; clypeus, mandibles, palpi, dorsal surface and dorso-posterior corner of pronotum, latero-anterior corners of mesoscutum, tegula, dorso-anterior corner of mesopleuron, scutellum, and mid and hind tarsi yellow. Wings hyaline, veins and pterostigma brown or blackish-brown.

**Male.** General structure and color similar to female, except general color blackish-brown (Fig. 12D–F); whole face, ventral markings of mesopleuron, fore and mid coxae, and anterior 1/2 of T3 yellow; yellow markings on lateral anterior corners of mesoscutum more distinct and extend posteriorly through notauli (Fig. 12E, F). Male genitalia with gonostyle broad, tapered and rounded apically (Fig. 11P, Q).

HW/HL = 1.9; OD = 0.25 mm, POL/OD = 0.5, OOL/OD = 0.7, POL/OOL = 0.7; FW/FH = 1.6, CLW/CLH = 2.5, MSL/BMW = 0.3; flagellum with 42 segments; average ratio of basal five flagellomeres length 2.6: 1.4: 1.2: 1.3: 1.0; MSSL/MSSW = 1.2; SCL/SCW = 1.1; maximum axis of propodeal spiracles 1.0 × as minimum axis; average ratio of hind tarsomere length 4.3: 2.1: 1.6: 1.0: 1.0; fore wing length 9.6 mm; RMI = 0.7; BNI = 0.3; hind wing length 6.7 mm; NI = 3.0; distal hamuli 7–8; T1 3.0 × as long as posterior width, 6.2 × as long as anterior width, 1.7 × as long as length of T2; T2 1.0 × as long as posterior width, 1.5 × as long as anterior width.

##### Bionomics.

This species has been collected from mountainous areas in Taiwan above 2100 m by Malaise trap. Hosts are unknown.

##### Distribution.

Taiwan (Nantou).

##### Etymology.

The specific name *pseudozangicus* is derived from the name of the species most morphologically similar to this new species, *A. zangicus* Sheng, Sun & Li, 2020. The name is an adjective.

##### Remarks.

Sexual dimorphism was observed in this species, with body color generally reddish-brown in females and brown in males. Additionally, the yellow markings on the ventral mesopleuron and metasomal tergites are present in males but absent in females. The amplification of *COI* sequences in this species failed in this study.

#### ﻿ Alexeter rufispeculus

Taxon classificationAnimaliaHymenopteraIchneumonidae

Chen, Huang & Shiao sp. nov.

E67E4508-455D-594F-816A-1ECA66892E68

https://zoobank.org/CBB6A622-5D4E-4CDC-BDBB-C7E29133CAAA

**Figure 9.**
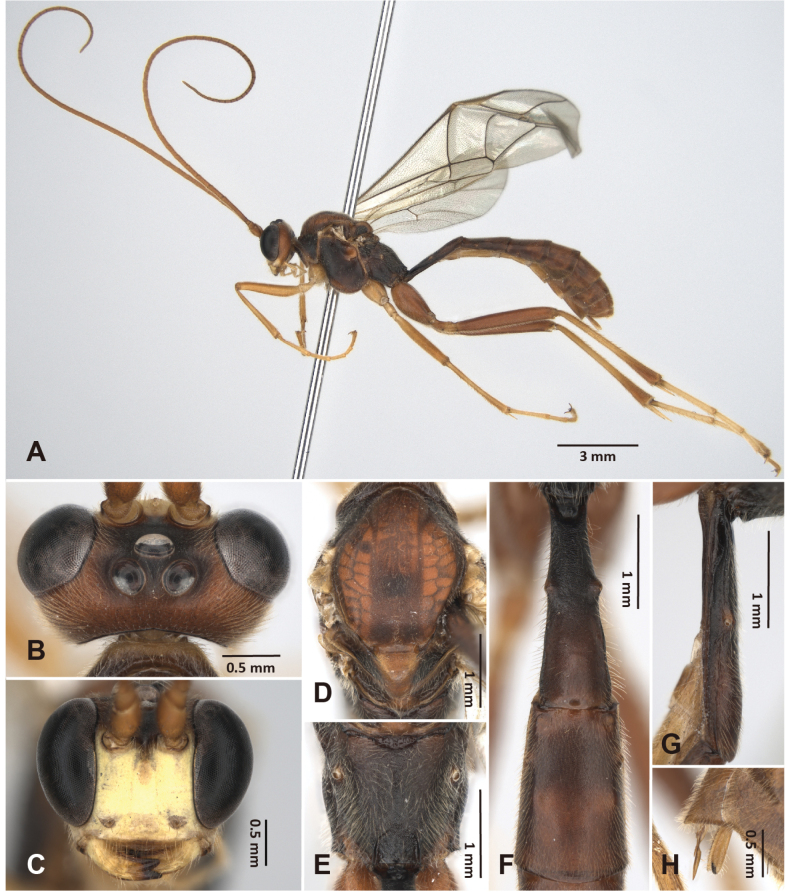
*Alexeter rufispeculus* sp. nov., holotype, black-type female (NMNS 8951-5; Mesolei08). **A.** Lateral habitus; **B.** Head in dorsal view; **C.** Head in anterior view; **D.** Mesoscutum in dorsal view; **E.** Propodeum in dorsal view; **F.** Metasomal tergites 1 and 2 in dorsal view; **G.** Metasomal tergite 1 in lateral view; **H.** Ovipositor.

**Figure 10.**
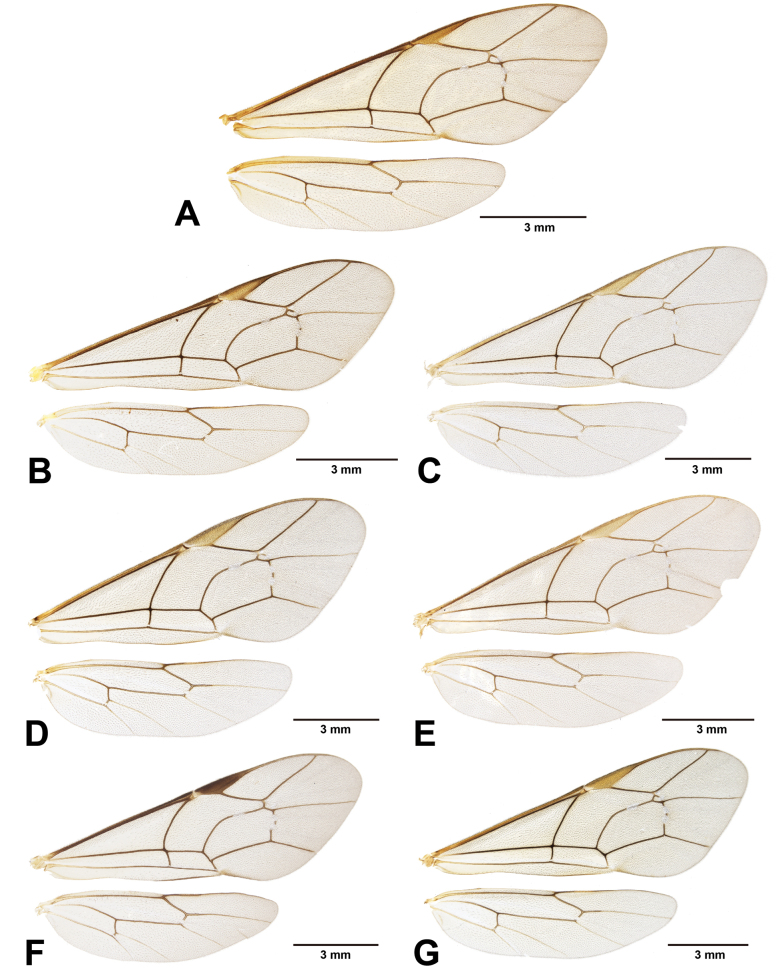
Wings of the Taiwanese *Alexeter* species. **A.***A. shakojiensis*; **B.***A. flavomaculatus* sp. nov.; **C.***A. hsiaoae* sp. nov.; **D.***A. mediolobus* sp. nov.; **E.***A. monticola* sp. nov.; **F.***A. pseudozangicus* sp. nov.; **G.***A. rufispeculus* sp. nov.

**Figure 11.**
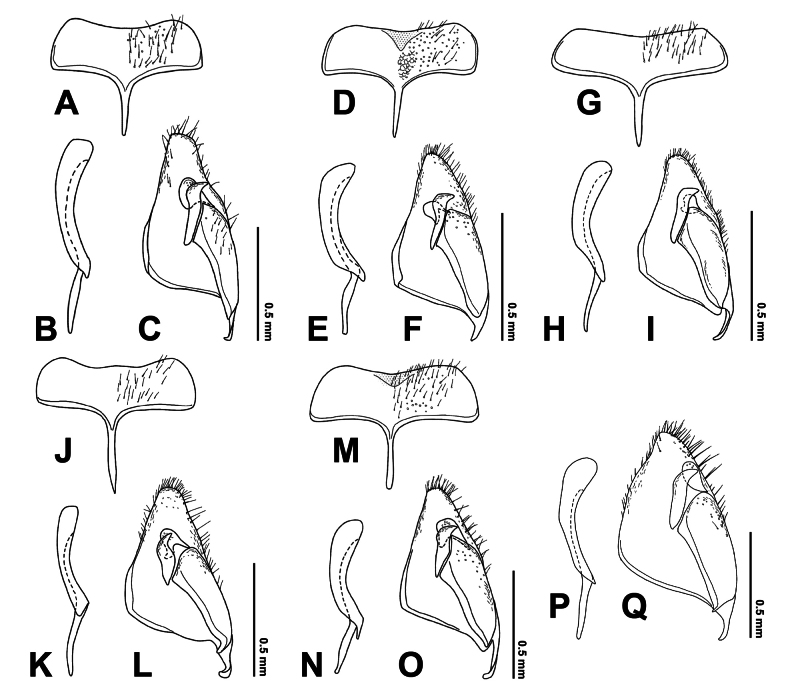
Male genitalia of the Taiwanese *Alexeter* species. **A–C.***A. shakojiensis*; **D–F.***A. flavomaculatus* sp. nov.; **G–I.***A. hsiaoae* sp. nov.; **J–L.***A. mediolobus* sp. nov.; **M–O.***A. rufispeculus* sp. nov.; **P, Q.***A. pseudozangicus* sp. nov.; **A, D, G, J, M.** Abdominal sternite 9 (posterior in upper portion); **B, E, H, K, N, P.** Penisvalva; **C, F, I, L, O, Q.** Right gonostyle in inner view.

**Figure 12.**
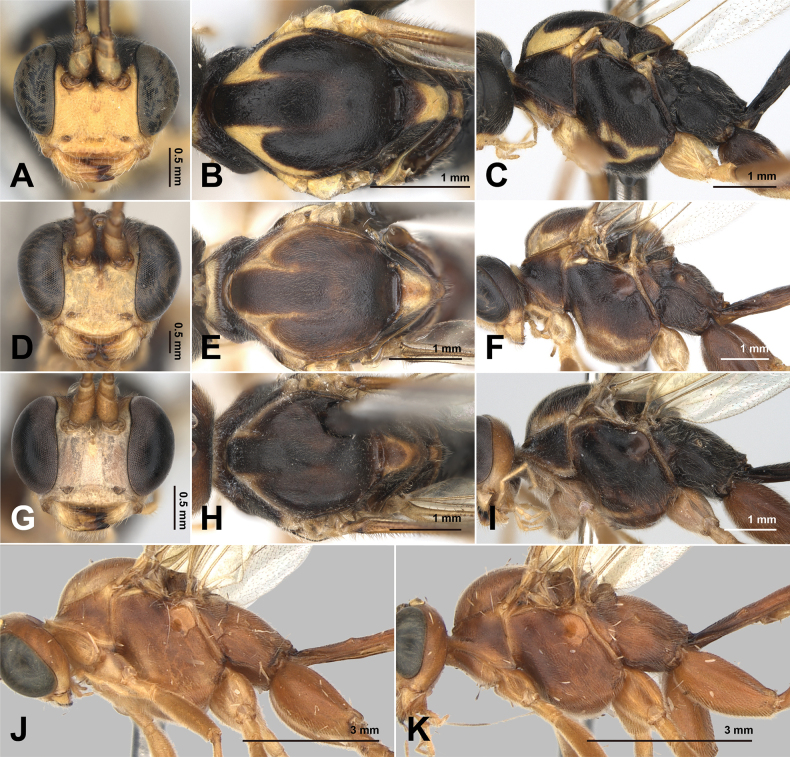
Color variations of the Taiwanese *Alexeter* species. **A–C.***A. flavomaculatus* sp. nov., male (TARI); **D–F.***A. pseudozangicus* sp. nov., male (TARI); **G–K.***A. rufispeculus* sp. nov., black-type male (**G–I**), red-type male (**J**), and red-type female (**K**) (TARI). **A, D, G.** Head in anterior view; **B, E, H.** Mesoscutum in dorsal view; **C, F, I, J, K.** Mesosoma in lateral view.

Figs 9A–H, 10G, 11M–O, 12G–K; Suppl. material 3 Chinese vernacular name: 鏡紅亞力姬蜂

##### Material examined.

***Holotype.*** Taiwan • 1♀; Yilan County, Datong Township, Siyuan Pass 42.0K (Quri Sqabu); 25. May. 2020; Light Trap; C. L. Huang & L. H. Wang leg.; GenBank: PV223409 (*COI*); NMNS ENT 8951-5 (Mesolei08). ***Paratypes.*** Taiwan • 1♀; Yilan County, Datong Township, Siyuan Pass (Quri Sqabu); 1. Jun. 2020; Light Trap; H. Y. Lee leg.; GenBank: PV223407 (*COI*); KPMNH (Mesolei04) • 1♂; Nantou County, Xinyi Township, Tatajia, Tai-18^th^ highway 100.5K, 23.480555°N, 120.852519°E (DD); alt. 2350 m; 20. Jun. 2020; Light Trap; C. L. Huang leg.; GenBank: PV223408 (*COI*); NMNS ENT 8951-6 (Mesolei09) • 1♂; Nantou County, Ren’ai Township, Meifeng; 23–24. Sep. 1997; Light Trap; C. S. Lin & W. T. Yang leg.; NMNS ENT 2692-791 • 9♀♀14♂♂; ibid; Jun. 1984; alt. 2150 m; Malaise Trap; K. S. Lin & K. C. Chou leg.; TARI (measure01–03) • 1♀9♂♂; ibid; May. 1984; TARI (measure04–05) • 3♀♀; ibid; Jul. 1984; TARI • 1♀1♂; ibid; 22–26. Jun. 1983; K. S. Lin & S. C. Lin leg.; TARI • 1♂; ibid; 24–26. Jun. 1981; K. S. Lin & W. S. Tang leg.; TARI • 1♀; ibid; 2–4. Jun. 1980; alt. 2130 m; L. Y. Chou & C. C. Chen leg.; TARI • 1♀; Nantou County, Ren’ai Township, Tsuifeng; alt. 2300 m; 5. Aug. 1984; K. S. Lin leg.; TARI (measure06) • 1♀; ibid; Sep. 1984; Malaise Trap; K. S. Lin & K. C. Chou leg.; TARI (measure07) • 1♀2♂♂; ibid; Aug. 1984; TARI (measure08–09) • 1♀; Nantou County, Ren’ai Township, 820 Forest Road; 25. Jun. 2008; Light Trap; H. H. Lin leg.; NMNS ENT 5880-85 • 1♀; Nantou County, Ren’ai Township, Tayuling, 820 Forest Road; 14. Jul. 2008; Light Trap; H. H. Lin leg.; NMNS ENT 6070-1565 • 1♀; ibid; 11. Aug. 2008; NMNS ENT 6070-1580 • 1♀; ibid; 3. Sep. 2008; NMNS ENT 6070-1608 • 1♀; Nantou County, Ren’ai Township, Piluchi Workstation; alt. 2100 m; 24–25. Jun. 2008; Light Trap; W. T. Yang leg.; NMNS ENT 5881-473 • 1♀; Nantou County, Ren’ai Township, Rueiyanxi, Shueiguan Road; 29–30. Aug. 2009; Light Trap; H. H. Liang leg.; NMNS ENT 6214-311 • 1♀; “Lishan” (= Taichung City, Heping Dist., Lishan); 12–18. Jul. 1971; Malaise Trap; Unknown collector; TARI • 3♀♀; Chiayi County, Alishan Township, Mt. Alishan; alt. 2400 m; 17–20. Aug. 1982; K. C. Chou & C. C. Pan leg.; TARI (measure10–11) • 1♂; ibid; 5–9. Aug. 1981; L. Y. Chou & S. C. Lin leg.; TARI (measure12) • 2♂♂; “Arishan” (= Chiayi County, Alishan Township, Mt. Alishan); Jun. 1914; M. Maki leg.; TARI (measure13) • 1♀; Hualien County, Xiulin Township, Tayuling; alt. 2560 m; 24–26. Jun. 1977; K. S. Lin leg.; TARI • 1♀; ibid; 9–16. Jun. 1980; K. S. Lin & B. S. Chen leg.; TARI.

##### Diagnosis.

This species can be distinguished from congeners by the combination of the following characters: ocelli large (OD = 0.20–0.30 mm; OOL/OD = 0.6–0.9); POL/OOL = 0.6–1.1; clypeus truncate on ventral margin (Figs 9C, 12G); fore wing areolet triangular with stalk, receiving 2m-cu at distal corner (Fig. 10G); fore wing vertical, basad, opposite, or distad to M&RS (Fig. 10G); lateromedian longitudinal carinae of propodeum present posteriorly with the area petiolaris opened (Fig. 10E); posterior transverse carina present (Fig. 10E); general body color variable from reddish-brown (red type) (Fig. 12J, K) to blackish-brown (black type) (Figs 9, 12G–I); speculum on the mesopleuron usually reddish-brown, rarely not (Figs 9A, 12I–K); males with yellow markings on the latero-anterior corners of mesoscutum (Fig. 12H–J).

The red-type male of this new species is similar to the female of *A. pseudozangicus* sp. nov. which shares the reddish-brown body color and yellow markings on the latero-anterior corners of the mesoscutum but differs in the following characters: clypeus truncate (Figs 9C, 12G) (rounded in *A. pseudozangicus*); mandible with both teeth equal in length (Figs 9C, 12G) (lower tooth slightly longer than upper tooth in *A. pseudozangicus*); areolet present (Fig. 10G) (absent in *A. pseudozangicus*); lateromedian longitudinal carinae of propodeum absent on the anterior and median portions (Fig. 9E) (complete in *A. pseudozangicus*); T1 3.0–4.3 × its posterior width (2.3–3.0 × in *A. pseudozangicus*); and the width of male gonostyle normal (Fig. 11O) (broad in *A. pseudozangicus*).

##### Description.

The measurements were based on Taiwanese specimens (15 females and 8 males).

**Female.** Head (Figs 9A–C, 12G): matt and granulate, HW/HL = 1.7–2.0 (1.9, 1.8 ± 0.11); ocelli large, with OD = 0.20–0.30 (0.26, 0.26 ± 0.03) mm, POL/OD = 0.4–0.8 (0.4, 0.5 ± 0.10), OOL/OD = 0.6–0.8 (0.7, 0.7 ± 0.06), POL/OOL = 0.6–1.1 (0.6, 0.8 ± 0.13); face matt and granulate, FW/FH = 1.2–1.5 (1.5, 1.4 ± 0.08); clypeus smooth with sparce punctures, truncate, and having transverse median ridge on ventral margin, CLW/CLH = 2.6–4.3 (3.7, 3.1 ± 0.43); MSL/BMW = 0.4–0.5 (0.4, 0.4 ± 0.04); mandible evenly punctate in dorsal surface, teeth equal in length or with lower tooth slightly longer than upper tooth; flagellum with 41–51 (49) segments; average ratio of basal five flagellomeres length 2.5: 1.3: 1.1: 1.1: 1.0.

Mesosoma (Figs 9A, D, E, 12H–K): polished and granulate; pronotum with epomia absent, weakly rugose on dorso-anterior corner; mesoscutum with MSSL/MSSW = 1.1–1.3 (1.1, 1.2 ± 0.06), notauli short, distinct on anterior ~0.2; scutellum with SCL/SCW = 0.9–1.4 (1.4, 1.1 ± 0.15), lateral carina present anteriorly; epicnemial carina weak, ~1/2 as height of mesopleuron; metapleuron with pleural carina and submetapleural carina complete; juxtacoxal carina vestigial posteriorly; propodeum with spiracle circular to suboval, maximum axis 1.0–1.3 (1.0, 1.1 ± 0.09) × as minimum axis; anterior transverse carina absent; posterior transverse carina present medially; lateromedian longitudinal carinae absent on anterior and median portions, present on posterior ~0.25 with area petiolaris opened anteriorly; lateral longitudinal carinae absent; average ratio of hind tarsomere length 4.3: 2.2: 1.7: 1.0: 1.2.

Wings (Fig. 10G): fore wing length 8.4–10.9 (10.7, 10.0 ± 0.66) mm; areolet triangular with stalk 0.3–0.6 (0.4, 0.5 ± 0.09) as long as 2rs-m, receiving 2m-cu at distal corner; RMI = 0.6–0.9 (0.7, 0.7 ± 0.08); 1cu-a vertical, basad, opposite, or distad to M&RS, with BNI = 0.1–0.3 (0.2, 0.2 ± 0.05). Hind wing length 6.2–8.2 (8.0, 7.5 ± 0.51) mm; NI = 1.6–3.0 (1.7, 2.2 ± 0.44); distal hamuli 6–8 (6).

Metasoma (Fig. 9F–H): polished and minutely coriaceous; T1 3.0–4.2 (3.3, 3.2 ± 0.31) × as long as posterior width, 5.8–9.5 (6.7, 7.2 ± 1.06) × as long as anterior width, 1.3–1.7 (1.4, 1.4 ± 0.09) × as long as length of T2; T1 with latero-median carina absent, dorso-lateral carina and ventro-lateral carina complete and varied from weak to strong, spiracle at around middle of T1, glymma distinct; T2 1.1–1.5 (1.5, 1.3 ± 0.09) × as long as posterior width, 1.6–2.3 (2.0, 1.8 ± 0.17) × as long as anterior width, gastrocoeli shallow and indistinct, thyridia circular; ovipositor sheath 2.4–5.5 (5.5, 3.6 ± 0.88) × as long as its maximum width in lateral view, shorter than apical depth of metasoma.

Color (Figs 9, 10G, 12K): variable, head, and legs generally reddish-brown, mesosoma and metasoma generally blackish-brown or brown in black-type specimens (including holotype) and reddish-brown in red-type specimens, except antenna, fore legs except tarsi, mid coxae, and trochanters yellowish-brown; face, clypeus (whole or in dorsal 1/2), mandibles, malar space, palpi, tegula, all tarsi, hind tibiae (whole or at least in basal 0.6) yellow or pale yellow; frons, ocellar area, ventral marking of clypeus and T1 (in some specimens) blackish-brown; speculum and scutellum always reddish-brown; and metasomal tergites behind T3 reddish-brown, sometimes tinged with red. Wings hyaline, veins blackish-brown or yellowish-brown, pterostigma pale yellowish-brown.

**Male.** General structure and color similar to female, except latero-anterior margin of mesoscutum and ventral side of mesopleuron with pale yellow marking (Fig. 12G–J). Male genitalia with gonostyle tapered and rounded apically, S9 weakly concave on posterior margin, with subtriangular median area weakly sclerotized (Fig. 11M–O).

HW/HL = 1.6–2.0 (1.8 ± 0.18); OD = 0.24–0.28 (0.26 ± 0.01) mm, POL/OD = 0.5–0.7 (0.6 ± 0.08), OOL/OD = 0.6–0.9 (0.7 ± 0.08), POL/OOL = 0.6–1.0 (0.8 ± 0.14); FW/FH = 1.2–1.4 (1.3 ± 0.05), CLW/CLH = 2.7–3.2 (3.0 ± 0.2), MSL/BMW = 0.2–0.4 (0.3 ± 0.05); flagellum with 43–49 segments; average ratio of basal five flagellomeres length 2.3: 1.2: 1.1: 1.1: 1.0; MSSL/MSSW = 1.2–1.3 (1.2 ± 0.06); SCL/SCW = 1.0–1.2 (1.1 ± 0.06); maximum axis of propodeal spiracles 1.0–1.1 (1.0 ± 0.06) × as minimum axis; average ratio of hind tarsomere length 4.1: 2.2: 1.7: 1.0: 1.1; fore wing length 8.6–10.1 (9.5 ± 0.52) mm; RMI = 0.6–0.9 (0.7 ± 0.12); BNI = 0.1–0.2 (0.2 ± 0.04); hind wing length 6.5–7.5 (7 ± 0.41) mm; NI = 1.5–3.7 (2.4 ± 0.75); distal hamuli 6–8; T1 2.9–4.1 (3.5 ± 0.34) × as long as posterior width, 5.8–8.0 (7.0 ± 0.73) × as long as anterior width, 1.3–1.6 (1.5 ± 0.08) × as long as length of T2; T2 1.3–1.6 (1.4 ± 0.13) × as long as posterior width, 1.8–2.4 (2.0 ± 0.2) × as long as anterior width.

##### Bionomics.

This species has been collected from mountainous areas in Taiwan above 2000 m by Malaise trap or light trap. Hosts are unknown.

##### Distribution.

Taiwan (Yilan, Hualien, Nantou, Taichung, and Chiayi).

##### Etymology.

The specific name *rufispeculus* is derived from the Latin words *rufi*- (meaning red) and *speculus* (meaning mirror, refer to speculum on the mesopleuron herein), referring to the reddish-brown speculum on the mesopleuron of this new species. The name is an adjective.

##### Remarks.

According to the collection data in this study, this is the most common species of *Alexeter* in Taiwan (Suppl. material 1). Large color variation was observed in this species, with two color types, black type (body color generally blackish-brown) (Figs 9, 12G–I) and red type (reddish-brown) (Fig. 12J, K). Both types have speculum on the mesopleuron reddish-brown (except one observed specimen in this study) (Figs 9A, 12I–K). Additionally, the sexual dimorphism was also observed in this species, with yellow markings on the latero-anterior corners of mesoscutum present in males (Fig. 12H–J) but absent in females (Figs 9A, D, 12K).

As mentioned in the Remarks of *A. mediolobus* sp. nov. above, endosymbiont co-amplification was also observed in this new species when amplifying the *COI*-5P region of the *COI* gene, with the same solutions applied. It is nested within *Alexeter* Clade I in the current *COI*-based phylogeny (Fig. 2).

## ﻿Discussion

### ﻿Evaluation of the species delimitation results

To describe biodiversity on Earth, biologists strive to connect species delimitation methods with species concepts to better understand the speciation process (de Queiroz 2007). In this study, we incorporate multiple lines of evidence by integrating morphological data, monophyly of *COI*-based molecular phylogeny, clusters of two DNA-based species delimitation algorithms (ASAP and bPTP), and the elevational distribution to the best of our ability to delimit the Taiwanese *Alexeter*.

In our *COI*-based species delimitation results, the optimal ASAP (ASAP #1) and the bPTP partitions are deemed implausible, since they proposed the *Alexeter* Clade I as single species exhibiting extreme morphological variation and a broad geographical distribution. For example, according to the reference lateral habitus photos available from the BOLD systems (see links in Suppl. material 1), the BIN BOLD:AGA9683 comprises at least three morphospecies: individuals with black heads and reddish-brown body color (e.g., ICHFI1878-16 and COLHH520-18), black body and reddish-brown T2–4 (e.g., ICHFI1870-16), and entirely black (e.g., COLHH2406-18). Similarly, BINsBOLD:AAH8583, BOLD:ADR4543, and BOLD:ACR4573 include individuals with generally black bodies and reddish-brown T2–4, and BOLD:AAW1857 includes individual with a black vertex, yellowish-white gena, and yellowish-brown body color (e.g., COLHH2401-18). The sampled Taiwanese species, *A. hsiaoae* sp. nov., *A. mediolobus* sp. nov., *A. rufispeculus* sp. nov., and *A. flavomaculatus* sp. nov., also display distinct morphological characters respectively. These morphologically distinct samples spanning from Taiwan, Europe, and Canada (Suppl. material 1), are oddly clustered as single species in optimal ASAP and bPTP partitions (Fig. 2). Therefore, despite the *COI* threshold distance in the suboptimal partition of ASAP (ASAP #2) being distinctively lower (K2P distance = 0.008414) than the commonly applied criteria for insects (K2P threshold distance = 0.03; Hebert et al. 2004), we prefer the species hypothesis from this partition due to its alignment with morphological evidence (Fig. 2). Given that *COI* sequences could not be successfully amplified for some of the species described herein, morphological characters were chosen as the primary criterion for species delimitation, allowing consistent application across all species, and DNA-based species delimitation results are treated as supporting evidence. For the newly described species included in the *COI*-based molecular phylogeny (Fig. 2), the results further support the morphological delimitation, as each of the four species is recovered as monophyletic group corresponding to its respective morphology.

### ﻿Incongruences between the molecular and morphological criteria

The incongruence between morphological and *COI*-based species delimitations observed in this study may be attributed to two possible explanations: the limitations of the *COI* barcode, and the shortcomings of morphological diagnostic characters in *Alexeter*. First, the limitations of the *COI* gene as a DNA barcode include interference from endosymbionts (e.g., *Wolbachia* and *Rickettsia*) (e.g., Klopfstein et al. 2016), nuclear mitochondrial DNA segments (NUMTs or pseudogenes) (e.g., Song et al. 2008; Chen et al. 2024b), and co-amplification of DNA from related organisms (e.g., Chen et al. 2024b), all of which may lead to inaccurate species delimitation. In this study, we ruled out the effects of the latter two issues (see Materials and methods); however, the DNA of endosymbionts was co-amplified in *A. mediolobus* sp. nov. and *A. rufispeculus* sp. nov., thus we cannot rule out the possibility that their *COI* sequences have been distorted by such interference, resulting in the low *COI* divergence within *Alexeter* Clade I, as reported in other Darwin wasps (Klopfstein et al. 2016). On the other hand, it may indicate that the morphological diagnostic characters used in the genus *Alexeter* require further revision, as multiple distinct morphospecies are clustered with extremely low *COI* divergence (e.g., BIN BOLD:AGA9683). However, since the four new species sampled in the *COI* analysis can be clearly distinguished by either coloration or structural differences and are recovered as monophyletic groups respectively, combined with the unavailability for evaluating all specimens of *Alexeter* Clade I in this study, we retain the species hypothesis based on morphological criteria. Therefore, the application of the *COI* barcode for species delimitation in this genus requires further evaluation through broader sampling and consideration of the aforementioned issues.

### ﻿Non-monophyly of the genus *Alexeter*

As for the non-monophyly of the genus *Alexeter* revealed herein (Fig. 2), it could be attributed to the limitation of the DNA marker and the possible misidentifications in the online databases. Despite the relatively high mutation rate of the *COI* gene, which makes it effective for species delimitation and species-level phylogenetics (e.g., Klopfstein et al. 2010; Quicke et al. 2012; Schwarzfeld et al. 2016), the nuclear marker *28S* is more commonly used in higher-level phylogenetic studies of Ichneumonoidea due to its better resolution above the genus level (e.g., Laurenne et al. 2006; Quicke et al. 2009). Therefore, relying solely on *COI* for molecular phylogenetic analyses may lead to biased or inconsistent results, particularly at higher taxonomic ranks.

Misidentifications are also common in online databases (e.g., Shimizu et al. 2020), especially when there is a lack of modern taxonomic studies for the target group. For instance, a Canadian BIN BOLD:AAH8583 has been incorrectly identified as “*A. coxalis* (Brischke, 1871)”, a European species. Given the vague generic boundaries between *Alexeter* and its closely related genera (see Taxonomic section above), it is possible that the sequences labeled as “*Alexeter*” in GenBank and BOLD systems include multiple genera, which probably accounts for the observed polyphyly of *Alexeter*.

### ﻿Biogeographical implications from the discovery of Taiwanese *Alexeter*

The discovery of the genus *Alexeter*, including one known species and six new species from the mid- to high-elevation mountains of Taiwan, confirms our expectation of its occurrence and indicates the underestimated mesoleiine fauna in Taiwan. A similar distribution pattern, with relatives of Holarctic taxa also present in Oriental mountainous areas, has been observed in other insects (e.g., Yeh et al. 2022; Chen et al. 2024a). Taiwan’s mountainous areas, with climates similar to those of temperate zones, offer diverse habitats and serve as refuges for species undergoing post-glacial recolonization and fostering insular endemism (e.g., beech trees and their associated herbivores (Hsu et al. 2021); *Hynobius* salamanders (Li et al. 2011)). These findings highlight the importance of conserving the montane fauna of Ichneumonidae in Taiwan, calling for further studies on their faunistic investigation, biogeographical origins, and ecology.

## Supplementary Material

XML Treatment for Alexeter

XML Treatment for Alexeter shakojiensis

XML Treatment for Alexeter flavomaculatus

XML Treatment for Alexeter hsiaoae

XML Treatment for Alexeter mediolobus

XML Treatment for Alexeter monticola

XML Treatment for Alexeter pseudozangicus

XML Treatment for Alexeter rufispeculus

## ﻿Additional information

### Use of AI

No use of AI was reported.

### Author ORCIDs

Hsuan-Pu Chen https://orcid.org/0000-0002-5605-5153

Shiuh-Feng Shiao https://orcid.org/0000-0002-4213-0423

### Data availability

All of the data that support the findings of this study are available in the main text or Supplementary Information.

## Supplementary materials

Supplementary material 1 Sample information of this study Data type xlsx Explanation note Spreadsheet containing the information of the sampled sequences and specimens. https://binary.pensoft.net/file/1407303This dataset is made available under the Open Database License (http://opendatacommons.org/licenses/odbl/1.0/). The Open Database License (ODbL) is a license agreement intended to allow users to freely share, modify, and use this Dataset while maintaining this same freedom for others, provided that the original source and author(s) are credited.Hsuan-Pu Chen, Chia-Lung Huang, Shiuh-Feng Shiao

Supplementary material 2 Sequence alignment and partition files used in this study Data type rar Explanation note The compressed .rar file containing the alignment of the *COI* dataset in Fasta format, as well as the best-scheme partition and substitutional models for phylogenetic reconstruction in Nexus format used in this study. https://binary.pensoft.net/file/1407304This dataset is made available under the Open Database License (http://opendatacommons.org/licenses/odbl/1.0/). The Open Database License (ODbL) is a license agreement intended to allow users to freely share, modify, and use this Dataset while maintaining this same freedom for others, provided that the original source and author(s) are credited.Hsuan-Pu Chen, Chia-Lung Huang, Shiuh-Feng Shiao

Supplementary material 3 Comparative tables of measurements, meristic counts, and characters Data type docx Explanation note The comparative tables include measurements and meristic counts of Taiwanese species of *Alexeter* and characters of *Alexeter* species of the world with yellowish- and reddish-brown body color. https://binary.pensoft.net/file/1407305This dataset is made available under the Open Database License (http://opendatacommons.org/licenses/odbl/1.0/). The Open Database License (ODbL) is a license agreement intended to allow users to freely share, modify, and use this Dataset while maintaining this same freedom for others, provided that the original source and author(s) are credited.Hsuan-Pu Chen, Chia-Lung Huang, Shiuh-Feng Shiao
