# Supplementary material for: ﻿A taxonomic revision of the genus Alexeter Förster (Hymenoptera, Ichneumonidae, Ctenopelmatinae, Mesoleiini) from Taiwan, with descriptions of six new species
Source: Zookeys. 2025 Sep 2;1250:315–58. doi: 10.3897/zookeys.1250.156835 (PMC12421253; doi:10.3897/zookeys.1250.156835)
Supplement: Supplementary material 3 — Comparative tables of measurements, meristic counts, and characters [file zookeys-1250-315_article-156835__-s003.docx]

**Table S1.** Comparison of the measurements, ratios, and meristic counts between the female *Alexeter* species from Taiwan. Abbreviations: L, length; W, width; pW, posterior width; aW, anterior width; n, numbers of measured specimens; N/A, unavailable; others as per materials and methods.

| Measurements | *Alexeter shakojiensis* | *Alexeter flavomaculatus* sp. nov. | *Alexeter hsiaoae* sp. nov. | *Alexeter mediolobus* sp. nov. | *Alexeter monticola* sp. nov. | *Alexeter pseudozangicus* sp. nov. | *Alexeter rufispeculus* sp. nov. |
| --- | --- | --- | --- | --- | --- | --- | --- |
|  | *n =* 8 | *n =* 1 | *n =* 3 | *n =* 6 | *n =* 10 | *n =* 6 | *n =* 15 |
| HW/HL | 1.8–2.1 | 1.8 | 1.6–1.9 | 1.7–2.1 | 1.6–1.8 | 1.6–1.8 | 1.7–2.0 |
| OD (mm) | 0.19–0.26 | 0.19 | 0.20–0.25 | 0.20–0.27 | 0.21–0.29 | 0.19–0.25 | 0.20–0.30 |
| POL/OD | 0.5–0.9 | 0.8 | 0.5–0.9 | 0.6–0.7 | 0.6–0.8 | 0.6–1.0 | 0.4–0.8 |
| OOL/OD | 1.0–1.4 | 1.4 | 0.7–0.9 | 0.5–0.7 | 0.5–0.7 | 0.8–1.1 | 0.6–0.8 |
| POL/OOL | 0.5–0.7 | 0.6 | 0.8–1.1 | 1.1–1.5 | 0.8–1.2 | 0.7–1.1 | 0.6–1.1 |
| FW/FH | 1.5–1.8 | 1.6 | 1.4 | 1.4–1.6 | 1.3–1.6 | 1.4–1.7 | 1.2–1.5 |
| CLW/CLH | 2.6–3.1 | 2.6 | 2.9–3.3 | 3.0–3.5 | 3.0–3.8 | 2.8–3.3 | 2.6–4.3 |
| MSL/BMW | 0.4–0.6 | 0.5 | 0.3–0.7 | 0.3–0.4 | 0.4–0.5 | 0.3–0.5 | 0.4–0.5 |
| Flagellum segments | 44–51 | 45 | 46 | 40–43 | 46–52 | 42–48 | 41–51 |
| MSSL/MSSW | 1.3–1.4 | 1.2 | 1.1–1.2 | 1.1–1.2 | 1.1–1.3 | 1.2–1.3 | 1.1–1.3 |
| SCL/SCW | 1.0–1.3 | 1.3 | 1.2–1.3 | 1.0–1.4 | 0.9–1.4 | 1.0–1.4 | 0.9–1.4 |
| PSI | 1.0–1.3 | 1.1 | 1.0–1.1 | 1.0–1.2 | 1.0–1.1 | 1.1–1.3 | 1.0–1.3 |

To be continued

**Table S1.** Continued.

| Measurements | *Alexeter shakojiensis* | *Alexeter flavomaculatus* sp. nov. | *Alexeter hsiaoae* sp. nov. | *Alexeter mediolobus* sp. nov. | *Alexeter monticola* sp. nov. | *Alexeter pseudozangicus* sp. nov. | *Alexeter rufispeculus* sp. nov. |
| --- | --- | --- | --- | --- | --- | --- | --- |
| Fore wing L (mm) | 9.7–11.9 | 8.3 | 8.7–10.4 | 7.7–10.1 | 10.7–12.0 | 10.1–12.2 | 8.4–10.9 |
| Areolt stalk L/2rs-m L | 0.4–0.6 | 0.5 | 0.4–0.5 | 0.3–0.4 | 0.3–0.5 | N/A | 0.3–0.6 |
| RMI | 0.6–0.9 | 0.7 | 0.5–0.8 | 0.6–0.7 | 0.6–1.0 | 0.7–0.9 | 0.6–0.9 |
| BNI | 0.1–0.3 | 0.2 | 0.1–0.2 | 0.1–0.2 | 0.1–0.2 | 0.2–0.3 | 0.1–0.3 |
| Hind wing L (mm) | 7.0–9.0 | 6.0 | 6.8–7.9 | 5.6–7.4 | 8.0–9.0 | 7.4–8.8 | 6.2–8.2 |
| NI | 1.3–2.1 | 2.2 | 1.7–3.0 | 1.9–2.8 | 1.8–3.7 | 2.3–4.3 | 1.6–3.0 |
| Distal hamuli | 6–9 | 6–7 | 5–6 | 6–8 | 6–9 | 7–8 | 6–8 |
| T1 L/pW | 2.7–3.3 | 3.5 | 3.1–3.8 | 2.5–3.1 | 3.4–3.9 | 2.3–2.8 | 3.0–4.2 |
| T1 L/aW | 7.2–8.7 | 6.6 | 6.2–8.2 | 5.8–8.0 | 6.7–8.2 | 5.8–7.0 | 5.8–9.5 |
| T1 L/T2 L | 1.4–1.5 | 2.3 | 1.3–1.5 | 1.4–1.5 | 1.4–1.6 | 1.5–1.7 | 1.3–1.7 |
| T2 L/pW | 1.2–1.5 | 0.8 | 1.1–1.4 | 0.9–1.3 | 1.2–1.5 | 0.9–1.2 | 1.1–1.5 |
| T2 L/aW | 1.7–2.4 | 1.3 | 1.9–2.3 | 1.4–2.1 | 1.9–2.4 | 1.3–1.5 | 1.6–2.3 |
| Ovipositor sheath L/W | 3.3–6.2 | 6.0 | 3.3–5.6 | 2.7–5.5 | 2.3–3.3 | 3.1–4.6 | 2.4–5.5 |

**Table S2.** Comparison of the measurements, ratios, and meristic counts between the male *Alexeter* species from Taiwan. Abbreviations: L, length; W, width; pW, posterior width; aW, anterior width; n, numbers of measured specimens; N/A, unavailable; others as per materials and methods.

| Measurements | *Alexeter shakojiensis* | *Alexeter flavomaculatus* sp. nov. | *Alexeter hsiaoae* sp. nov. | *Alexeter mediolobus* sp. nov. | *Alexeter pseudozangicus* sp. nov. | *Alexeter rufispeculus* sp. nov. |
| --- | --- | --- | --- | --- | --- | --- |
|  | *n =* 4 | *n =* 3 | *n =* 7 | *n =* 1 | *n =* 1 | *n =* 8 |
| HW/HL | 1.7–2.1 | 1.8–1.9 | 1.7–1.9 | 1.9 | 1.9 | 1.6–2.0 |
| OD (mm) | 0.22–0.26 | 0.15–0.19 | 0.18–0.23 | 0.25 | 0.25 | 0.24–0.28 |
| POL/OD | 0.6–0.7 | 0.5–0.9 | 0.6–0.9 | 0.6 | 0.5 | 0.5–0.7 |
| OOL/OD | 1.0–1.2 | 1.2–1.6 | 0.6–0.8 | 0.5 | 0.7 | 0.6–0.9 |
| POL/OOL | 0.5–0.7 | 0.4–0.6 | 0.9–1.1 | 1.3 | 0.7 | 0.6–1.0 |
| FW/FH | 1.6–1.8 | 1.4–1.8 | 1.3–1.7 | 1.5 | 1.6 | 1.2–1.4 |
| CLW/CLH | 2.9–3.2 | 3.0–4.0 | 3.0–3.6 | 2.9 | 2.5 | 2.7–3.2 |
| MSL/BMW | 0.4–0.5 | 0.5 | 0.3–0.4 | 0.4 | 0.3 | 0.2–0.4 |
| Flagellum segments | 46–51 | 43–45 | 42–47 | N/A | 42 | 43–49 |
| MSSL/MSSW | 1.3–1.5 | 1.2–1.3 | 1.1–1.3 | 1.1 | 1.2 | 1.2–1.3 |
| SCL/SCW | 0.9–1.2 | 1.1–1.2 | 1.0–1.4 | 1.2 | 1.1 | 1.0–1.2 |
| PSI | 1.0–1.1 | 1.0–1.2 | 1.0–1.2 | 1.1 | 1.0 | 1.0–1.1 |

To be continued

**Table S2.** Continued.

| Measurements | *Alexeter shakojiensis* | *Alexeter flavomaculatus* sp. nov. | *Alexeter hsiaoae* sp. nov. | *Alexeter mediolobus* sp. nov. | *Alexeter pseudozangicus* sp. nov. | *Alexeter rufispeculus* sp. nov. |
| --- | --- | --- | --- | --- | --- | --- |
| Fore wing L (mm) | 10.0–11.0 | 7.2–7.8 | 7.8–9.7 | 8.9 | 9.6 | 8.6–10.1 |
| Areolt stalk L/2rs-m L | 0.4–0.8 | 0.4–0.6 | 0.3–0.5 | 0.4 | N/A | 0.3–0.6 |
| RMI | 0.7–0.8 | 0.7–0.8 | 0.6–0.8 | 0.7 | 0.7 | 0.6–0.9 |
| BNI | 0.1–0.3 | 0.1–0.2 | 0.1–0.2 | 0.2 | 0.3 | 0.1–0.2 |
| Hind wing L (mm) | 7.1–7.8 | 5.2–5.6 | 5.7–7.0 | 6.6 | 6.7 | 6.5–7.5 |
| NI | 1.4–2.2 | 2.0–2.7 | 1.8–3.0 | 1.9 | 3.0 | 1.5–3.7 |
| Distal hamuli | 7–10 | 6–7 | 4–7 | 6 | 7–8 | 6–8 |
| T1 L/pW | 2.8–3.7 | 2.7–3.1 | 3.5–4.3 | 2.9 | 3.0 | 2.9–4.1 |
| T1 L/aW | 7.3–8.1 | 6.1–6.6 | 7.0–10.4 | 8.9 | 6.2 | 5.8–8.0 |
| T1 L/T2 L | 1.4–1.6 | 1.3–1.6 | 1.3–1.5 | 1.5 | 1.7 | 1.3–1.6 |
| T2 L/pW | 1.3–1.6 | 1.2–1.4 | 1.2–1.6 | 1.3 | 1.0 | 1.3–1.6 |
| T2 L/aW | 1.9–2.4 | 1.6–1.9 | 1.6–2.6 | 1.7 | 1.5 | 1.8–2.4 |

**Table S3.** Comparative table of yellowish- or reddish-brown species of *Alexeter* of the world. Abbreviations: OR, Oriental region; WP, Western Palearctic region; EP, Eastern Palearctic region; NE, Nearctic region; pW, posterior width; N/A, unavailable; others as per materials and methods.

|  | *A. hsiaoae* sp. nov. | *A. mediolobus* sp. nov. | *A. monticola* sp. nov. | *A. clavator* (Müller, 1776) | *A. nebulator* (Thunberg, 1822) | *A. gracilentus* (Holmgren, 1857) | *A. luteifrons* (Cresson, 1868) |
| --- | --- | --- | --- | --- | --- | --- | --- |
| Distribution | OR | OR | OR | WP, EP | WP, EP | WP, EP | NE |
| References | this study | this study | this study | photos;  Sheng et al. (2020) | this study;  Sheng et al. (2020) | Lectotype photos (♀) | Holotype photos (♂) |
| OOL/OD | 0.7–0.9 | 0.5–0.7 | 0.5–0.7 | ~0.6–0.8 | ~0.7–0.9 | 0.9 | ~1.0 |
| POL/OOL | 0.8–1.1 | 1.1–1.5 | 0.8–1.2 | ~1.3 | ~0.8–1.0 | 0.7 | ~0.9 |
| Mandibles | teeth equal in length | teeth equal in length | teeth equal in length | teeth equal in length | teeth equal in length | lower tooth longer | N/A from photos |
| MSL/BMW | 0.3–0.7 | 0.3–0.4 | 0.4–0.5 | ~0.15 (China); ~0.5 (UK) | ~0.4 | 0.6 | ~0.4 |
| Flagellum | 46 (♀); 42–47 (♂) | 40–43 (♀); N/A (♂) | 46–52 (♀); N/A (♂) | 46 (♀); 40–44 (♂) | 50–51 (♀); 44–46 (♂) | broken | N/A from photos |
| Fore wing length (mm) | 7.8–10.4 | 7.7–10.1 | 10.7–12.0 | 9.5–11.0 | 9.5–11.0 | ~9.3 | ~6.8 |
| Areolet | present; trapezoid with stalk | present; trapezoid with stalk | present; triangular or trapezoid with stalk | present; triangular with stalk | present; triangular with stalk | present; triangular with stalk | present; trapezoid with stalk |
| 1cu-a | vertical, opposite or slightly distad M&RS | vertical and opposite M&RS | vertical and almost opposite M&RS | almost vertical and distad M&RS | vertical and slightly distad M&RS | almost vertical and distad M&RS | vertical and opposite M&RS |

To be continued

**Table S3. Continued.**

|  | *A. hsiaoae* sp. nov. | *A. mediolobus* sp. nov. | *A. monticola* sp. nov. | *A. clavator* (Müller, 1776) | *A. nebulator* (Thunberg, 1822) | *A. gracilentus* (Holmgren, 1857) | *A. luteifrons* (Cresson, 1868) |
| --- | --- | --- | --- | --- | --- | --- | --- |
| Lateromedian longitudinal carina | present only in posterior portion | present only in posterior portion | present only in posterior portion | vestigial in anterior and median portions | vestigial in anterior and median portions | vestigial in anterior and median portions | N/A from photos |
| Area periolaris | opened | almost closed | opened | almost closed | opened | opened | N/A from photos |
| Lateral longitudinal carina | vestigial posteriorly | complete but weak | vestigial posteriorly | distinct | vestigial posteriorly | vestigial posteriorly | N/A from photos |
| Posterior transverse carina | vestigial medially | distinct medially | absent | distinct medially | vestigial medially | vestigial medially | N/A from photos |
| T1 L/pW | 3.1–4.3 | 2.5–3.1 | 3.4–3.9 | ~3.3 | 3.2–3.6 | ~4.1 | N/A from photos |
| Color | | | | | | | |
| Face and clypeus | yellowish-white | yellowish-white | yellowish-white | yellowish-brown | yellow to yellowish-brown | yellow | yellow |
| Temples | yellowish-white | yellowish-brown | yellowish-brown | reddish-brown | black | black | black |
| Vertex | brown to blackish-brown | brown to blackish-brown | yellowish-brown to orange | reddish-brown | black | black | black |
| Gena | yellowish-white | yellowish-brown | yellowish-brown to orange | reddish-brown | yellowish-brown | yellow | yellow |

To be continued

**Table S3.** Continued

|  | *A. hsiaoae* sp. nov. | *A. mediolobus* sp. nov. | *A. monticola* sp. nov. | *A. clavator* (Müller, 1776) | *A. nebulator* (Thunberg, 1822) | *A. gracilentus* (Holmgren, 1857) | *A. luteifrons* (Cresson, 1868) |
| --- | --- | --- | --- | --- | --- | --- | --- |
| Coloration | | | | | | | |
| Pronotum | yellowish-brown | yellowish-brown | yellowish-brown to orange | reddish-brown | yellowish-brown | reddish-brown, brown dorsally | reddish-brown |
| Mesoscutum | yellowish-brown with three distinct blackish-brown stripes on lobes | brown with single blackish-brown stripe on median lobe | yellowish-brown to orange, with three blackish-brown stripes on lobes | entirely reddish-brown | yellowish-brown, male with yellow markings on latero-anterior corners | entirely reddish-brown | entirely reddish-brown |
| Scutellum | yellowish-white | brown | yellowish-brown | reddish-brown | yellowish-brown | yellow | yellow |
| Mesopleuron | pale yellowish-brown | brown | yellowish-brown | reddish-brown | yellowish-brown, male with yellow marking ventrally | reddish-brown, with yellow marking ventrally | reddish-brown, with yellow marking ventrally |
| Metasoma | yellowish-brown, brown in T1 | brown | brown or yellowish-brown | reddish-brown | yellowish-brown | blackish-brown in T1 and T2, tinged with reddish-brown apically | reddish-brown |
| Legs | yellowish-brown | yellowish-brown | yellowish-brown | reddish- or yellowish-brown | yellowish-brown | yellowish-brown | yellowish-brown |
